# *AaJAZ8* forms an extensive interaction network with *AaJAZ* proteins and two novel *AaMYC* transcription factors in *Artemisia annua*

**DOI:** 10.3389/fpls.2026.1855917

**Published:** 2026-06-11

**Authors:** Xianchun Zong, Chaoxue Ma, Baosheng Liao

**Affiliations:** 1 College of Life Science and Technology, Mudanjiang Normal University, Mudanjiang, Heilongjiang, China; 2 The Second Clinical College, Guangzhou University of Chinese, Medicine, Guangzhou, Guangdong, China

**Keywords:** *Artemisia annua*, JAZ, MeJA, protein-protein interaction, stress response

## Abstract

*Artemisia annua* is the source plant for artemisinin production. Variable cultivation conditions are important factors affecting plant growth and artemisinin yield. The jasmonic acid signaling pathway is a crucial mechanism by which plants cope with stress, with the jasmonate ZIM-domain (*JAZ*) genes playing key roles in this pathway. While the *JAZ* gene family in *A. annua* has not yet been comprehensively characterized, this study conducted an in-depth analysis of *JAZ* genes, including identification and molecular characteristics such as genetic variation and alternative splicing, as well as short-term expression responses to methyl jasmonate (MeJA) in the LQ-9 genotype. The results showed that 18 *AaJAZ* genes were identified in the h0 haplotype, whereas 17 were identified in the h1 haplotype. A total of 967 single nucleotide polymorphisms (SNPs) and 267 insertion/deletion (indels) were identified across 17 pairs of alleles, and four *AaJAZ* genes showed alternative splicing events. Ten other Aa*JAZ* genes exhibited specific and significant induction by MeJA exclusively in old leaves. Through gene expression profiling and protein-protein interaction validation, AaJAZ8 was found to form homodimers and interact with 10 AaJAZs. Additionally, yeast two-hybrid assays suggested potential physical interactions between AaJAZ8 and both AaMYC24 and AaMYC26. This study systematically characterizes the JAZ protein family in *A. annua*, identified novel AaJAZ8-interacting proteins, and provides a foundation for further understanding the role of *AaJAZ8* in the jasmonate signaling network.

## 1 Introduction

*Artemisia annua* L., an annual herbaceous plant within the *Artemisia* genus of the *Asteraceae* family, serves as the source of artemisinin, a sesquiterpene lactone compound extensively utilized in malaria treatment. The plant growth and artemisinin biosynthesis of *A. annua* are substantially influenced by adverse environmental conditions (Tu et al., 1981; Zhang et al., 2023). During the long - term evolutionary process, plants have established a complex defense signaling network to address environmental stresses and pathogen attacks. Among these, jasmonates, recognized as crucial phytohormones, are widely regarded as core signaling molecules that regulate secondary metabolism (Ali and Baek, 2020). Research has demonstrated that the exogenous application of jasmonic acid can effectively activate the defense response in *A. annua*, significantly up - regulating the expression of key genes (e.g., *AaCYP71AV1* and *AaADS*) in the artemisinin biosynthetic pathway, thereby facilitating the accumulation of artemisinin (Hao et al., 2017; Li et al., 2019). Moreover, recent investigations suggest that the molecular mechanisms underlying jasmonic acid signaling and artemisinin synthesis vary between the young and mature leaves of *A. annua* (Fu et al., 2026).

The jasmonate ZIM-domain (JAZ) proteins serve as central transcriptional repressors within the jasmonic acid signaling process. The JAZ proteins are members of the plant-specific TIFY superfamily, which encompasses the TIFY, ZIM-like, PEAPOD, and JAZ protein subfamilies (Bai et al., 2011). Typically, the JAZ proteins possess two highly conserved sequence motifs: the JAZ proteins feature a conserved TIFY (also referred to as ZIM) domain that contains a core TIF[F/Y]XG motif in the vicinity of the N-terminal region (Vanholme et al., 2007); the C-terminal region harbors a JA-associated (Jas) domain, initially described previously (Yan et al., 2007), and is characterized by an S-L-X(2)-F-X(2)-K-R-X(2)-R core.

The majority of JAZ proteins are capable of forming homo-and heterodimers (Chung and Howe, 2009; Pauwels and Goossens, 2011; Xu et al., 2021), and the dimerization of JAZ proteins may be associated with the sensitivity to Methyl jasmonate (MeJA). Multiple transcription factors (TFs) interact with *JAZ* genes. MYC2, a positive regulator of JA signaling, was the first TF reported to be regulated by JAZ proteins (Chini et al., 2007). In *A.annua*, AabHLH1 interacts with nine AaJAZ (AaJAZ1-9) proteins (Li et al., 2019), and AaMYC2 can interact with four AaJAZ proteins (AaJAZ1-4) (Shen et al., 2016). Additionally, protein-protein interactions with JAZ proteins, as demonstrated by the yeast two-hybrid (Y2H) system, have been identified for COI1 (F-box receptor) (Thines et al., 2007), R2R3 MYB TFs (Kayani et al., 2023), and corepressor proteins (Pauwels et al., 2010). *AaJAZ8*, a core transcriptional repressor of jasmonic acid signaling in *A. annua*, attenuates the activities of *AaMYC2* (Ma et al., 2018), *AaYABBY5* (Kayani et al., 2023), *AaHD1* (Yan et al., 2017), *AaTCP14* (Ma et al., 2018), and *AaMYB108* (Liu et al., 2023), while AaJAZ9 participates in the negative regulation of *WRKY9* (Fu et al., 2021).

A plant genome may contain a considerable number of JAZ members (Chini et al., 2007; Song et al., 2022), and different JAZ members may exhibit distinct response mechanisms and regulatory roles. However, the JAZ members in *A. annua* have not yet been fully characterized. With the release of multiple *A. annua* genomes (Shen et al., 2018; Liao et al., 2022b), extenive analysis of *JAZ* genes has become feasible. In this study, leveraging the two haplotype genomes of *A. annua* LQ-9, we conducted a comprehensive analysis of the *JAZ* gene family, including identification, allelic variation, and alternative splicing. Leaves at different maturation stages were treated with MeJA and subjected to gene expression profiling. Furthermore, protein interactions between AaJAZ8 and related TFs, and dimerization of AaJAZ8 were investigated. This work led to the identification of two novel MYC genes capable of interacting with AaJAZ8, providing new scientific insights into the regulatory role of AaJAZ proteins in the jasmonic acid signaling pathway.

## 2 Materials and methods

### 2.1 Plant materials

This study used the LQ-9 strain of *A. annua*, which contains two haplotypes (h0 and h1). We chose LQ-9 for two main reasons. Our lab has plenty of LQ-9 tissue-cultured plantlets on hand; Our group already has a large amount of transcriptomic data for LQ-9, making it easier to integrate and compare with new data. For seed germination, we placed *A. annua* seeds on moist filter paper at 20 °C with 60% relative humidity in the dark. Once the seeds germinated, we selected seedlings of similar size and transplanted them into pots. These pots were then placed in a growth chamber set to 25 °C, with a light intensity of 12,000 lx and a 16-h light/8-h dark cycle. Three independent biological replicates were set up, each coming from a single source.When the plants reached the 7–9 leaf stage, they were classified into different groups according to leaf maturation stages: from the top to the bottom, leaves 1–3 were designated as the tender leaf group (TL), leaves 4–6 as the mature leaf group (ML), and leaves 7 and above as the old leaf group (OL).

### 2.2 Identification of JAZ members in *A. annua*

First, all the annotated proteins in the LQ - 9 haplotype 0 (h0) and LQ - 9 haplotype 1 (h1) genomes were subjected to a search against the PFAM database (Pfam 32.0) via PfamScan (E value ≤1e - 5) (http://www.ebi.ac.uk/Tools/pfa/pfamscan). Genes that exhibited hits to PFAM ID PF09425 (Jas domain) or PF06200 (TIFY/ZIM domain) were regarded as candidate *AaJAZ* genes. Second, the genes were visualized and rectified using the Apollo browser (Dunn et al., 2019) as previously detailed based on RNA - Seq and full - length transcripts (Wang et al., 2020). Third, to eliminate false - positive results, all the *AaJAZ* genes were re - verified using PfamScan. Finally, the entire set of *AaJAZ* genes of LQ - 9 h0 was searched against that of LQ - 9 h1 using BlastP (Camacho et al., 2009). The allelic (one-to-one) relationship among these *AaJAZ* genes was ascertained according to the amino acid sequence reciprocal best BLAST hits. The candidate *AaJAZ* gene sequences possessing TIFY or Jas domains were selected for further analysis.

### 2.3 Sequence information analysis

The number of amino acids, isoelectric points (pI), and molecular weights (MW) were computed using ProtParam (https://web.expasy.org/protparam/). The domains of *AaJAZ* genes were identified using PfamScan (http://pfam.xfam.org/). The duplicated *AaJAZ* genes were identified by the two criteria: (1) the protein length of the shorter sequence covered ≥75% of the longer sequence and (2) the similarity of the two aligned sequences was ≥90%. The subcellular localization of candidate AaJAZ proteins was predicted by DeepLoc - 2.0 (https://services.healthtech.dtu.dk/services/DeepLoc-2.0/).

Genetic variation detection: the genomic regions of gene body and 500 bp flanking sequences of the alleles were extracted. Alleles in LQ-9 h0 were used as references, and alleles in LQ-9 h1 were aligned to references with minimap2, followed by variant calling with paftools (https://github.com/lh3/minimap2/blob/master/misc/paftools.js). Variants were plotted using trackViewer (Ou and Zhu, 2019). Alternative splicing events were identified manually based on full length transcripts. All the sequences of *AaJAZ*s can be downloaded from GPGD: http://www.gpgenome.com/species/92.

### 2.4 Phylogenetic analysis of AaJAZ genes

A multiple sequence alignment of AaJAZ proteins in LQ-9 h0 and LQ-9 h1 was conducted using clustal-omega (Sievers et al., 2011). A maximum likelihood (ML) phylogenetic tree was constructed using RAxML (Stamatakis, 2014) with 1,000 bootstrap replicates. In addition, an ML phylogenetic tree containing JAZ proteins in *Arabidopsis thaliana* was built following the same process. Besides the 13 previously reported JAZ proteins, we also included the entire TIFY superfamily from *Arabidopsis* in the tree construction (TAIR10, https://www.arabidopsis.org/).

### 2.5 Validation of genetic variations and alternative splicing

To validate single nucleotide polymorphisms (SNPs) between alleles, two pairs of amplification primers were designed from conserved sequences of flanking regions of SNPs (**Supplementary Table 1**). Additionally, a pair of primers was designed for amplification of isoforms of *AaJAZ8* (**Supplementary Table 1**). The PCR amplification mixture consisted of 1 μL of cDNA, 10 μL of 2 × Taq Master Mix (Vazyme Biotech Co., Ltd), 0.4 μL of 10 μmol/L forward and reverse primers, and 8.2 μL of ddH_2_O. The PCR reactions included an initial denaturation step at 95 °C for 3 min, followed by 34 cycles: 30 s at 95 °C, 30 s at 55-60 °C, and 30–90 s at 72 °C, concluding with a holding step at 72 °C for 5 min. The PCR products used to detect allelic genetic differences were subjected to gel extraction and purification, followed by TA cloning using the 5 minTA/Blunt-Zero Cloning Kit (Vazyme Biotech Co., Ltd). Twenty randomly selected clones were then subjected to Sanger sequencing. The amplified products of isoforms were first separated by agarose gel electrophoresis and then sequenced. All the sequencing was conducted by Guangzhou IGE Biotechnology Co., Ltd.

### 2.6 MeJA treatment, RNA-Seq and expression profile analysis

TL, ML, and OL were selected from robust and disease - free plants. Two hours subsequent to spraying with 100 µmol/L MeJA (dissolved in 1% absolute ethanol), the leaves were sampled. The control group was treated with 1% absolute ethanol only. All samples were immediately flash-frozen in liquid nitrogen and stored at -80 °C for subsequent transcriptome sequencing. The total RNA extraction and quality-control of each sample were conducted using methods previously reported (Wang et al., 2025). Briefly, liquid nitrogen-milled samples were subjected to RNA extraction using the RNA kit (OMEGA, R6827-01) following the manufacturer instructions, and RNA samples with an RNA Integrity Number ≥ 7.5 were retained.

The RNA-Seq library construction and sequencing were carried out following protocols of MGI sequencing platform and approximate 8 Gb of 150-bp paired-end reads were generated for each sample. Library sizes varied across samples, with one sample (MeJA100_OL_3) having approximately twice the read depth of the others. To ensure that this imbalance did not bias the conclusions, we verified that DESeq2’s median-of-ratios normalization effectively accounts for library size differences. In addition, we confirmed that completely removing this sample from the analysis did not alter the OL-specific DEG patterns.

Raw read quality was evaluated using FastQC (https://www.bioinformatics.babraham.ac.uk/projects/fastqc/), and low - quality bases or reads were filtered out using fastp (Chen, 2025) with default parameters (**Supplementary Table 2**). The LQ - 9 h0 genome (Liao et al., 2022a) was downloaded from GPGD (Liao et al., 2022a) and used as the reference genome. Read alignment and gene quantification in different samples were conducted by HISAT2 and StringTie (Pertea et al., 2016). Hierarchical clustering analysis of expression levels was carried out using the “pheatmap” package in R. Differentially expressed genes (DEGs) were identified using the “DESeq2” package (Wang et al., 2010). DEGs were defined as genes that exhibited a fold change ≥ 2 and a BH - adjusted p - value (FDR) ≤ 1e - 6. All the RNA - Seq data can be downloaded from GPGD. http://www.gpgenome.com/species/92.

### 2.7 Gene functional enrichment analysis

Gene ontology (GO) enrichment analysis was carried out using clusterProfiler (Yu et al., 2012), and enrichment results with a value of *p ≤*1e-3 were retained.

### 2.8 Co-expression analysis of JAZ genes

Co-expression analysis was performed using the WGCNA pipeline (Langfelder and Horvath, 2008) in R as described below: The network type was set as unsigned, and a soft-thresholding power of β = 6 was selected. This value was the smallest value that gave a scale-free topology fit index R² above 0.9. A topological overlap matrix (TOM) was computed, followed by average-linkage hierarchical clustering. To detect initial modules, we applied the Dynamic Tree Cut method with a minimum module size of 30 and deepSplit = 2. Modules whose eigengene correlation exceeded 0.75 (corresponding to mergeCutHeight < 0.25) were merged, which yielded 12 modules for downstream analyses. Pearson’s correlation analysis was used to estimate the association between module eigengenes and treatment or different leaves.

### 2.9 Expression analysis by quantitative PCR

RNA samples for sequencing were further synthesized into cDNA. And actin was used as a reference gene. Primers for the *AaJAZ* genes (**Supplementary Table 3**) were designed and synthesized by Guangzhou IGE Biotechnology Co., Ltd. The qPCR reaction was performed using the LightCycler^®^96 Instrument (Roche, Switzerland). The PCR amplification mixture contained 1 μL of cDNA, 10 μL of ChamQ Universal SYBR qPCR Master Mix (Vazyme Biotech Co., Ltd), 0.4 μL of 10 μm forward and reverse primers, and 8.2 μL ddH_2_O. The PCR reaction was performed with the initial denaturation step for 3 min at 95 °C; 40 cycles of 3 s at 95 °C and annealing at 60 °C for 32 s, and a holding step for 30 s at 72 °C. The Student’s *t*-test was used to compare the means between two groups (*p* < 0.05).

### 2.10 Yeast two-hybrid assays

Full-length coding sequence of *AaJAZ8* was cloned into pGBKT7 vector between *Sfi*I and *Not*I as bait, and a total of 24 genes were inserted into pGADT7 vector, respectively (**Supplementary Figure 1**). Primers employed for amplification of genes as well as for vectors construction were listed in **Supplementary Table 4**. Each of the target pGADT7 vectors was co-transferred with pGBKT7-*AaJAZ8* into yeast strain Y2HGold, respectively. And transformed strains were cultured on SD/-Trp/-Leu plates. Interaction status was tested on SD/-Trp/-Leu and SD/-Trp/-Leu/-His/-Ade (+ 400 ng/mL AbA + X-α-gal). Y2HGold[pGBKT7-53+pGADT7-T] served as positive control strain, and Y2HGold[pGBKT7-Lam+pGADT7-T] served as negative control strain, and Y2HGold[pGBKT7-*AaJAZ8*+pGADT7] was used as a control to detect self-activation of the bait plasmid.

## 3 Results

### 3.1 Identification and characterization of the *AaJAZ* gene family in haplotype genomes of *A. annua*

**Figure 1:**
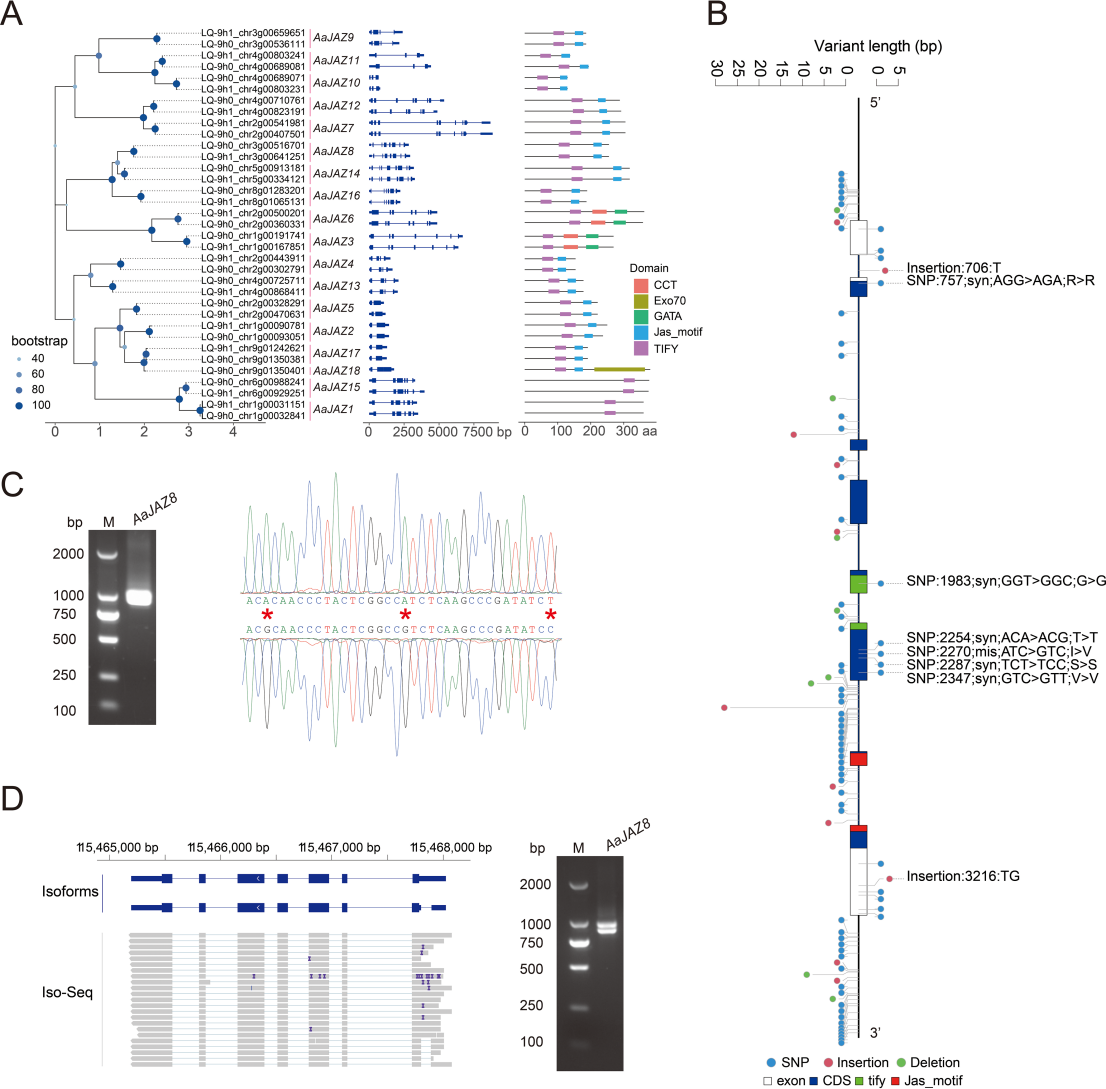
Characteristics of *AaJAZ* genes. **(A)** Phylogenetic relationships, gene structures, and domains of the alleles. Blue rectangles represent the coding sequences, thin blue lines connecting two exons represent introns, and thick blue lines represent 5’-UTR or 3’-UTR. **(B)** Genetic variations between *AaJAZ8* alleles. “syn” and “mis” represent synonymous and nonsynonymous, respectively. **(C)** Agarose gel electrophoresis and Sanger sequencing of *AaJAZ8* fragment containing SNPs. Red stars represent SNP loci. **(D)** Alternative splicing of *AaJAZ8* and validation using agarose gel electrophoresis. Iso-Seq represents the full-length transcripts.

A total of 18 *AaJAZ* genes were identified in h0, while 17 *AaJAZ* genes were identified in h1. All 17 *AaJAZ* members in h1 had one-to-one alleles in h0 (**Figure 1A**). Additionally, one *AaJAZ* in h0, chr9g01350381, had an extra copy named chr9g01350401 (**Supplementary Table 5**). Based on the order of *AaJAZ* genes on the chromosomes, the *AaJAZ* genes in *A. annua* were named as *AaJAZ1* to *AaJAZ18*. According to the phylogenetic analysis with *Arabidopsis JAZ* genes, the *AaJAZ* genes were divided into three clades (**Supplementary Figure 2**). Clade I contains 13 *AaJAZ* genes, all of which possess one TIFY and one Jas motif. Clade II contains two *AaJAZ* genes, both of which only have one TIFY domain. Clade III contains two *AaJAZ* genes, each containing one TIFY domain, one CCT domain, and one GATA domain (**Supplementary Figure 2**).

The lengths of the encoded amino acid sequences of *AaJAZ* genes spanned from 130 (*AaJAZ10)* to 380 (*AaJAZ18*), and the number of exons varied between two (*AaJAZ5/AaJAZ18*) and nine (*AaJAZ14*). Subcellular localization analysis revealed that all *AaJAZ* proteins are localized within the nucleus (**Supplementary Table 6**). Although predictions from nuclear localization signal analysis suggest that all JAZ proteins possess potential nuclear localization signals, a systematic validation of their subcellular localization through green fluorescent protein (GFP) fusion assays remains necessary. The protein sequence similarities between alleles ranged from 87.2% (*AaJAZ2*) to 100% (*AaJAZ3/AaJAZ11*). Moreover, significant genetic variations were detected among alleles: a total of 967 single - nucleotide polymorphism (SNP) sites and 267 insertions - deletions (indels) were identified across 17 pairs of alleles. Among these, 15.72% of the total SNP sites were situated in the coding sequence (CDS) region, and of these CDS SNPs, 7.34% resulted in amino acid Ch alterations. Similarly, 6.74% of the total indels occurred in the CDS region, and of these CDS indels, 1.50% led to frameshift mutations (**Supplementary Table 7**). Regions containing allelic variations were verified by polymerase chain reaction (PCR) amplification and Sanger sequencing (**Figures 1B, C**; **Supplementary Figure 3**).

Four *AaJAZ*s exhibited alternative splicing isoforms identified by full-length transcripts: intron retention and alternative 3’ splicing events of *AaJAZ7* yielded five splice variants; intron retention in 5’-UTR region of *AaJAZ8* yielded two splice variants; alternative 3’ splicing of *AaJAZ14* yielded three splice variants; and alternative 3’ splicing and intron retention in the 3’-UTR region of *AaJAZ15* yielded four splice variants (**Supplementary Figure 4**). Alternative splicing in the *AaJAZ7*, *AaJAZ14*, and *AaJAZ15* changed coding of amino acid sequences. The two distinct alternative splicing of *AaJAZ8* was experimentally validated using cDNA as a template (**Figure 1D**).

### 3.2 Gene expression profiles of different maturation stage leaves in response to MeJA

**Figure 2:**
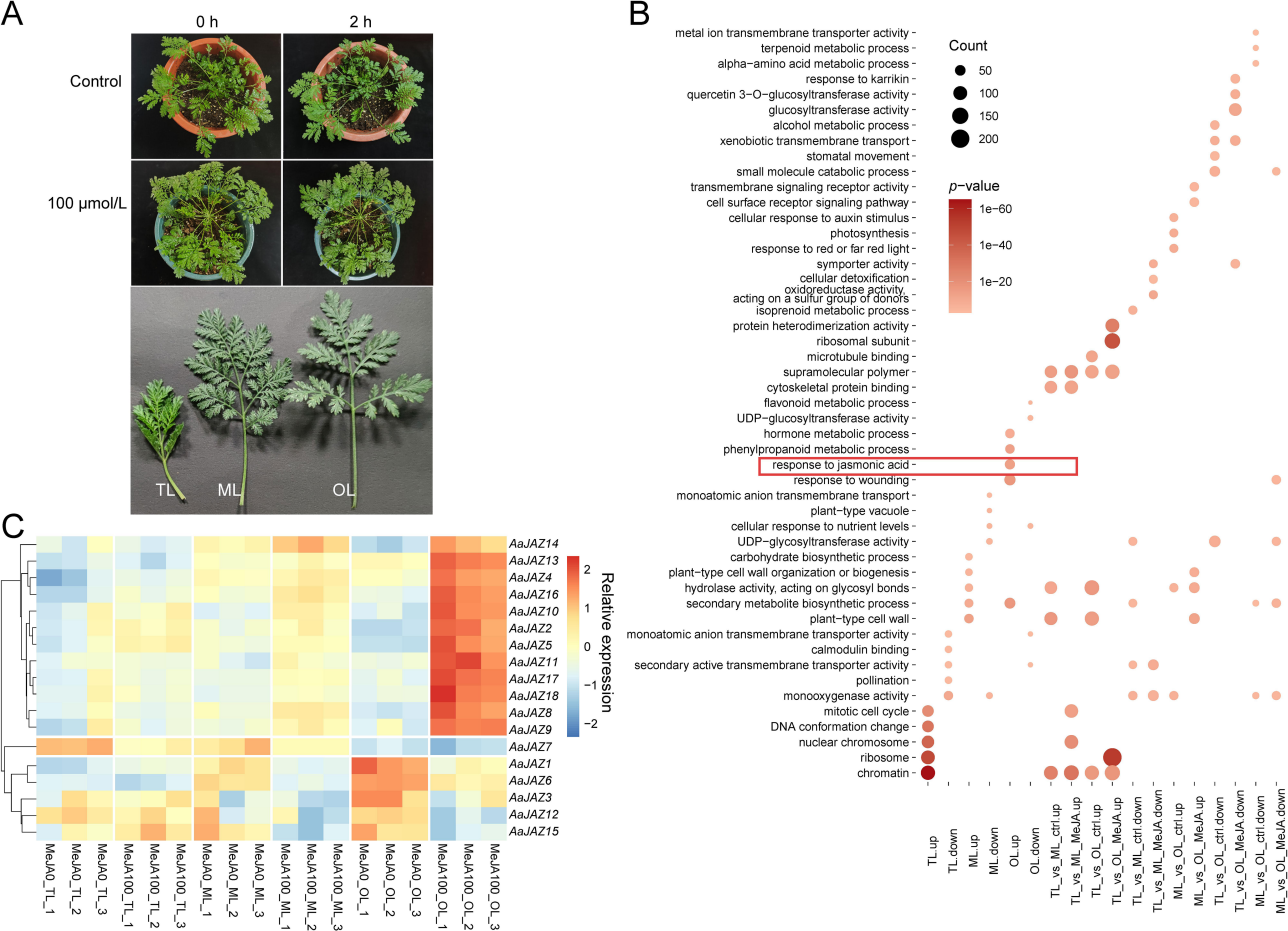
MeJA treatment and RNA-Seq analysis of *A. annua* leaves at different maturation stages. **(A)**The upper pot images show: left, before MeJA treatment (0 h); right, after 2 h of MeJA treatment. The lower panel shows the morphology of leaves at different developmental stages. TL, tender leaves; ML, mature leaves; OL, old leaves **(B)** Functional enrichment of differentially expressed genes. Top five Gene Ontology terms were shown. “ctrl” represents control sample, and “MeJA” represent sample treated with 100 µmol/L MeJA. **(C)** *AaJAZ*s Heatmap. “MeJA100” represents treatment with 100 µmol/L MeJA, and “MeJA0” represents the control.

JAZ proteins are widely recognized as key components that respond to and function within the jasmonic acid signaling pathway (Pauwels and Goossens, 2011). To reveal the expression pattern of *AaJAZ*s in response to MeJA, a treatment experiment involving the exogenous addition of MeJA was carried out. Leaves at different maturation stages of *A. annua* were treated with 100 μmol/L MeJA for 2 hours, and no visible changes were observed in the phenotype (**Figure 2A**). Approximately 8 Gb of RNA-Seq data was generated for each sample (**Supplementary Table 2**). Compared to control samples, 1117 genes were up-regulated and 1104 genes were down-regulated in TL; 719 genes were up-regulated and 582 genes were down-regulated in ML; and 1139 genes were up-regulated and 535 genes were down-regulated in OL (**Supplementary Figure 5**). Overall, the number of specifically upregulated/downregulated genes in each type of leaf was significantly higher than the number of genes upregulated/downregulated shared by two or more types of leaves (**Supplementary Figure 5**). A total of 329 genes were up-regulated and 420 genes were down-regulated in two or more leaf types. Functional enrichment analysis of genes up- or down-regulated in any two or more leaf types revealed that up-regulated genes were primarily enriched in processes such as secondary metabolite biosynthetic process, phenylpropanoid/L-phenylalanine biosynthetic process, aromatic amino acid metabolic process, ammonia-lyase activity, and cinnamic acid biosynthetic/metabolic process (**Figure 2B**; **Supplementary Figure 6A**). In contrast, down-regulated genes were mainly enriched in cellular response to nutrient levels, monooxygenase activity, cellular response to starvation, and catalytic reactions involving the oxidation or reduction of sulfur atoms (**Figure 2B**; **Supplementary Figure 6B**). This indicates that upon MeJA stimulation, *A. annua* demonstrated a “defense over growth” response. Meanwhile, the upregulated genes in various leaf types also exhibited distinct functional enrichments (**Figure 2B**). As an illustration, the upregulated genes in TL compared to the control samples had a higher level of enrichment in ribosome and mitotic cell cycle, whereas those in ML were more enriched in cell wall organization or biogenesis. Interestingly, upregulated genes in OL were enriched in response to jasmonic acid. Different leaf types have been found to regulate different sets of genes, thereby implying that the responses to MeJA differed across leaves. It was shown that MeJA treatment had a strong impact on gene regulation of all the leaf types, but the jasmonic acid signaling pathway was highly stimulated in OL alone. Also, the leaves at various levels of maturation showed a different set of DEGs, and the composition of DEGs in the various types of leaves changed significantly with the treatment of MeJA (**Figure 2B**). More than half of the *AaJAZ* members have shown substantial responses to MeJA. All of the 11 *AaJAZ* genes had different levels of expression between different leaf types or upon MeJA treatment. Out of this, 10 *AaJAZ* genes were highly inducible by MeJA in OL (**Figure 2C**). The trends of expression of these ten *AaJAZ* genes as determined by qPCR were mostly found to be consistent with the RNA-Seq data (**Supplementary Figure 8**). Those genes encoding the SCF complex involved in the degradation of *AaJAZ* were significantly not induced at 2 hours of MeJA treatment (**Supplementary Figure 8**) indicating that proteasomal degradation of *AaJAZ* had not been initiated. The genes in ABP(Artemisinin Biosynthetic Pathway), which are downstream of the jasmonic acid signaling pathway and whose activation depends on JAZ degradation (Shen et al., 2016), were not found to be induced by short-term MeJA treatment (**Supplementary Figure 8**).

### 3.3 Identification of core co-expression network of *AaJAZ*s

**Figure 3:**
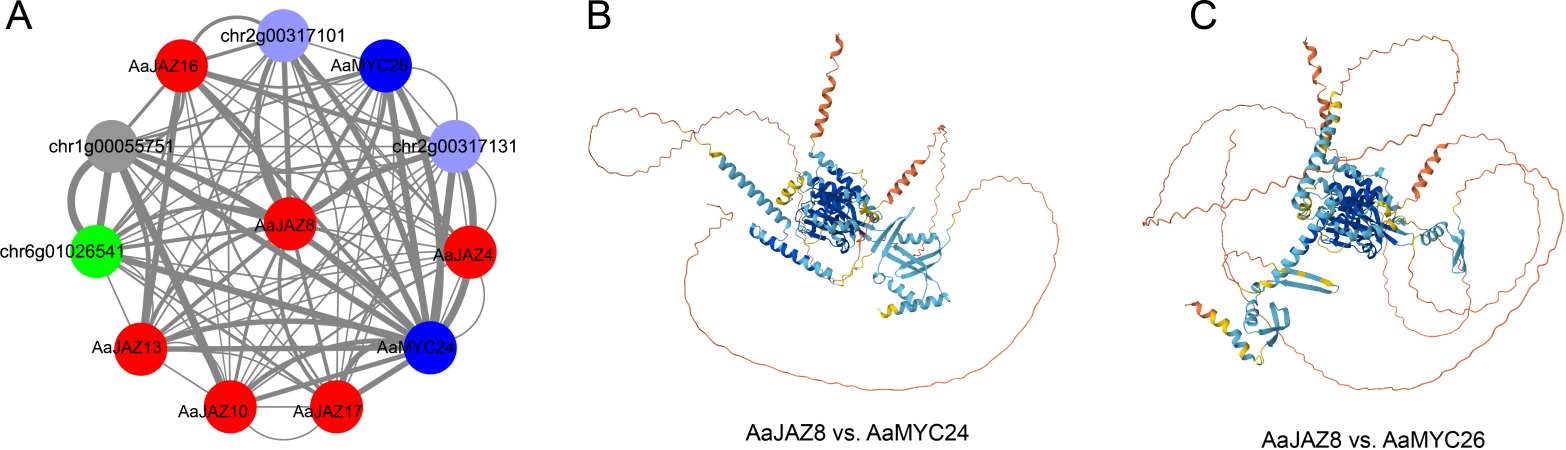
Co-expression network of AaJAZ8. Node colors represent different protein types: red, JAZ; blue, MYC; purple, bHLH; green, MYB; gray, zinc-finger RING. thick lines indicate strong interactions; thin lines indicate weak interactions **(A)** Predicted 3D structures of protein interactions between AaJAZ8 and two AaMYC proteins **(B, C)**. The ipTM scores are 0.50 and 0.43, respectively, both below the recommended confidence threshold of 0.6, indicating low-confidence interface predictions. Therefore, these structural models should be interpreted with caution and are presented for illustrative purposes only.

To identify potential interaction partners for the differentially expressed *AaJAZ*s, a co-expression network analysis was performed. The results showed that 13 modules were obtained, and 11 differentially expressed *AaJAZ*s were assigned into two modules. *AaJAZ2*, *AaJAZ4*, *AaJAZ5*, *AaJAZ8*, *AaJAZ10*, *AaJAZ11*, *AaJAZ13*, *AaJAZ14*, *AaJAZ16*, and *AaJAZ17* were in brown module and *AaJAZ7* was in salmon module. The brown module which containing 690 genes showed significantly positive correlation with MeJA treatment (**Supplementary Figure 9A**). Genes in the brown module were especially enriched in the function of response to jasmonic acid and secondary metabolisms biosynthetic process (**Supplementary Figure 9B**). By calculating the interconnectivity of genes within the brown module, gene pairs with an interconnectivity ≥ 0.3 were selected to construct a network using Cytoscape (Shannon et al., 2003). The results demonstrated high correlations between six AaJAZ proteins and six TFs (**Figure 3A**). These six TFs comprised two MYCs (chr2g00266181, unctg_3931g01664791), two bHLHs (chr2g00317101, chr2g00317131), one MYB (chr6g01026541), and one zinc finger (chr1g00055751). And all these six TFs were significantly induced by MeJA in the OL (**Supplementary Figure 10**). *AaJAZ8*, the core responder in jasmonic acid signaling in *A. annua*, was found to interact with multiple TFs, and among the MYCs, only *AaMYC2* has been experimentally validated (Ma et al., 2018). A total of 26 *AaMYC*s were identified in the LQ-9 h0 genome (**Supplementary Table 8**). Among them, nine *AaMYC*s exhibited induced expression under MeJA treatment, while only two *AaMYC*s, *AaMYC24* (chr2g00266181) and *AaMYC26* (unctg_3931g01664791), showed co-expression with *AaJAZ8*. Notably, *AaMYC2* (chr9g01406681) did not display significant differential expression in this study. In addition, *bHLH* and *MYB* are among the largest transcription factor families in plants and are widely involved in secondary metabolism, development, and stress responses. In *A. annua*, both types of transcription factors have been reported to participate in the regulation of artemisinin biosynthesis. Therefore, we hypothesized that two *bHLHs* (chr2g00317101 and chr2g00317131) and one *MYB* (chr6g01026541) may have functional associations with *AaJAZ8*.

We then used AlphaFold3 to compute the interactions between the AaJAZ8 protein and 11 other proteins, as well as its self-interaction. The top three Interface pTM scores were 0.51 (AaJAZ8 vs. AaJAZ10), 0.50 (AaJAZ8 vs. AaMYC24), and 0.43 (AaJAZ8 vs. AaMYC26) (**Supplementary Table 9**). The protein-protein interaction diagrams for AaJAZ8 with AaMYC24 and AaMYC26 are shown in **Figures 3B** and **C**.

### 3.4 Y2H assay of protein-protein interaction of AaJAZ8

**Figure 4:**
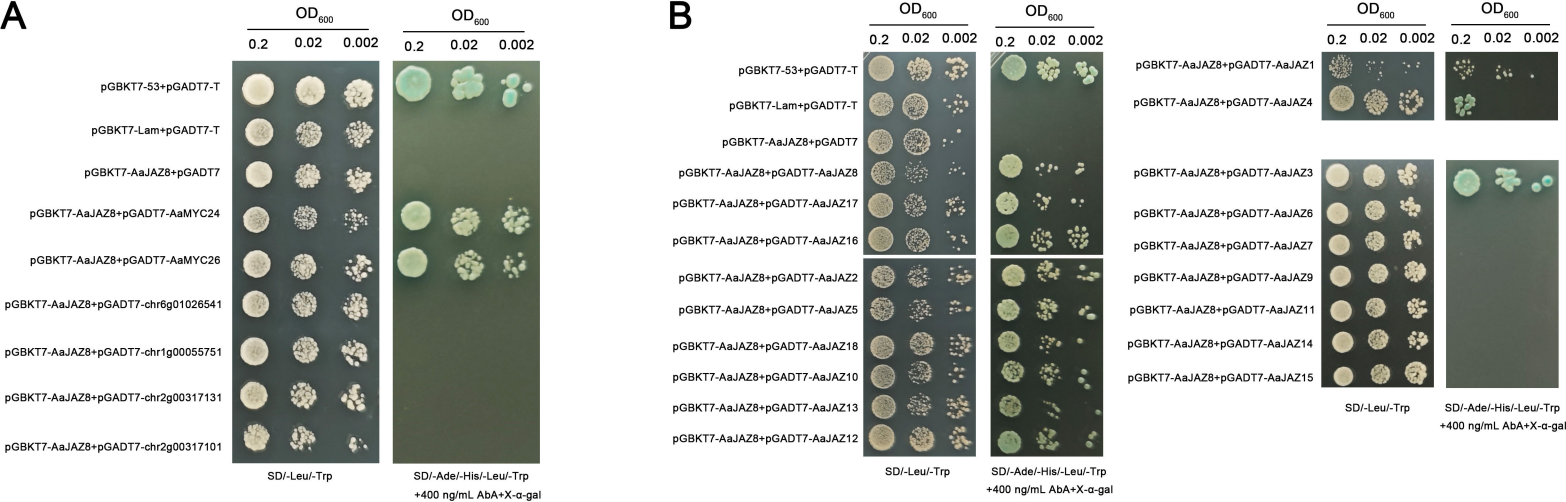
Verification of protein-protein interactions by Y2H. **(A)** Yeast two-hybrid screening of AaJAZ8-interacting proteins. chr2g00317101 and chr2g00317131 are bHLHs, chr6g01026541 is MYB, and chr1g00055751 is zinc finger. **(B)** Interaction between AaJAZ8 and other JAZs. The three independent transformation experiments were conducted with similar results; some images are provided as representatives. In order to determine interaction strength, co-transformed yeast cells were cultivated in SD/-Leu/-Trp and SD/-Leu/-Trp/-His/-Ade selection plates.

The yeast two - hybrid (Y2H) assay was conducted to examine the interactions between AaJAZ8 and each of the six TFs. The findings indicated that Y2HGold yeast strains co - transformed with AaMYC24/AaMYC26 and AaJAZ8 were capable of growing on SD/−Ade/−His/−Leu/−Trp medium supplemented with 400 ng/mL aureobasidin A (AbA) and X - α - gal, and formed blue colonies, which verified a physical interaction (**Figure 4A**; **Supplementary Table 10**). Conversely, no interaction was detected between AaJAZ8 and the other two basic helix - loop - helix (bHLH) proteins, one myeloblastosis (MYB) protein, and one zinc finger protein (**Figure 4A**; **Supplementary Table 10**).

To validate the homo - and heterodimeric interactions of AaJAZ8 with itself and other AaJAZ proteins, all 18 possible combinations between AaJAZ8 and each AaJAZ protein were tested using the Y2H assay. The results revealed that AaJAZ8 could form a homodimer with itself and heterodimers with ten other AaJAZ proteins (**Figure 4B**). Among these, the interactions with AaJAZ2, AaJAZ5, AaJAZ10, AaJAZ12, AaJAZ13, AaJAZ16, AaJAZ17, and AaJAZ18 were strong, while those with AaJAZ1 and AaJAZ4 were relatively weak. Notably, the Y2HGold strain co - transformed with AaJAZ1 and AaJAZ8 showed significantly weaker growth on SD/−Leu/−Trp plates compared to the other co - transformants. Nevertheless, this combination still grew on the more stringent SD/−Leu/−Trp/−His/−Ade plates, whereas the negative controls did not, suggesting a genuine but weak interaction rather than transformation toxicity.

## 4 Discussion

A total of 18 and 17 *AaJAZ*s were identified in the two haplotypes h0 and h1 of LQ-9, respectively. The additional *AaJAZ18* in h0 was tandem replicate of *AaJAZ17*. The number of *JAZ*s in *A. annua* is comparable to that in maize (16) (Han and Luthe, 2021) and sorghum (18) (Shrestha and Huang, 2022), but significantly less than in sunflower (27) (Song et al., 2022) and sugarcane (49) (Zhou et al., 2024). High levels of genetic variability were noted among allelic *AaJAZs*, and 7.34 percent of the SNPs and 1.5 percent of the indels changed the amino acid code, as the diversity of AaJAZs was demonstrated. This is also a weakness in this research as we have only tested the allelic differences in the genetics of *AaJAZ4* and *AaJAZ8* rather than all members of *JAZ1-17*. Further research is required to disassemble the genetic diversity and functional importance of the whole *JAZ* family. Moreover, four *AaJAZs* genes had alternative splicing isoforms increasing the variety of *AaJAZ* transcripts and encoded proteins and indicating the sophisticated transcriptional regulatory network of *AaJAZs*. An intron retention phenomenon was identified in the non-coding region of *AaJAZ8*. The sequence analysis confirmed the absence of change in the CDS sequence and the amino acid sequence encoded by it and it was expected that it would not be able to influence the interaction between proteins. However, this type of non-coding intron retention regulation of translation is still unresolved. Additional experiments using ribosome profiling (Ribo-seq) or dual-luciferase reporter should be conducted to examine this potential.

Different maturation stage leaves (TL, ML, OL) of *A. annua* treated by MeJA showed significant transcriptome reprogramming, where the total number of upregulated genes exceeded the number of downregulated genes in every leaf type. Commonly upregulated genes (i.e., commonly upregulated in two or more types of leaves) tended to have a greater enrichment in the biosynthetic pathways of secondary metabolites-particularly phenylpropanoids and aromatic amino acids, and commonly downregulated genes (downregulated in two or more leaf types) had a strong association with nutrient responses. These results implied that MeJA treatment generally prompts a defense over growth approach, wherein plants increase their stress tolerance by upregulating integrated defense-related metabolic pathways at the expense of other competing pathways. It is worth noting that (Yuan and Zhou, 2026) recently characterized *AaSPATULA*, a bHLH transcription factor that promotes both glandular trichome initiation and leaf development in *A. annua*, thereby linking vegetative growth to artemisinin yield. Based on these findings, future studies could explore whether JA treatment influences the vegetative growth of *A. annua* either positively or negatively, and how the trade-off between defense responses and growth might be balanced to optimize artemisinin production. Additionally, the various leaves demonstrated significantly different response patterns to MeJA, with each having its own distinctive set of DEGs. It is also important to note that the response to jasmonic acid signaling is leaf-age specific, the jasmonic acid response pathway was specifically and strongly induced in OL, which differs with the pattern of JA response decay with developmental progression in *Arabidopsi*s (Mao et al., 2017; Zhang et al., 2022). This discrepancy could indicate a difference in defense mechanisms between the two annual species: *Arabidopsis* has given up defense in senescent leaves in order to invest resources into reproduction, while *A. annua* OL have a higher MeJA response to offset the reduction in artemisinin biosynthesis in developing leaves or to control the production of old leaf-specific metabolites. In line with this, virtually all the differentially expressed *AaJAZs* were induced by MeJA in OL only, which further supports the specificity of *AaJAZs* to jasmonic acid signaling in relation to leaf age. An exception was *AaJAZ7*, which was highly expressed in TL but not in OL (**Figure 2C**), suggesting a TL-specific function in jasmonate signaling, possibly controlling trichome development or artemisinin biosynthesis. Considering the extremely different reactions to MeJA of leaves of different maturity, the careful choice of leaf material in different stages of development should be used in future studies with stress treatments and associated research. Nevertheless, it is worth mentioning that the OL-specific responses in the present study can be partly explained by inter-tissue variations in the basal activity of the jasmonate pathway, instead of the actual differential responsiveness to MeJA treatment. As we have not measured endogenous JA and JA-Ile concentrations in the untreated TL, ML and OL samples, we cannot entirely rule out this confounding factor. Subsequent studies are needed to measure basal JA and JA-Ile concentrations in different tissues using LC-MS to provide more accurate dissection of the nature of tissue-specific responses.

The *AaJAZs* were induced by MeJA in the 2 - hour time point in this study. Nevertheless, the genes of the SCF complex genes involved in the JAZ degradation were not significantly induced, which means that the JAZ degradation and the downstream pathway activation might happen at later steps. Likewise, long-term treatment of MeJA has been shown to upregulate genes that participate in the artemisinin biosynthesis process. As an illustration, Hao et al. found that when exposed to light, the level of *AaADS*, *AaCYP71AV1*, and *AaALDH1* were raised to 3.5-4.9 times the control after 8 hours of MeJA treatment, and it was found that *AaDBR2* expression increased to 7.1-fold (Hao et al., 2017). In another experiment, when exposed to MeJA for 0.5, 1, 3, 6, 9, 12, and 24 hours, *AaADS* expression was only highly upregulated at 6, 9, and 12 hours, with 12 hours being the highest time-point (Li et al., 2019). Also, Yuan et al. demonstrated that the MeJA-response transcription factor *AabHHLH113* was significantly elevated at 6 and 12 hours post-treatment (Yuan et al., 2023). In this paper, the MeJA treatment lasted just 2 hours, which could be insufficient to reach the minimum time of action necessary to activate the expression of downstream *ABP* genes, which can explain the lack of significant upregulation.

*MYC2* has been identified as the central TF in the jasmonic acid signaling pathway (Zhang et al., 2012). *AaMYC2* in *A. annua* is reported to be involved in jasmonic acid - mediated regulation. Under these circumstances, AaJAZ8 is capable of inhibiting *AaMYC2* function and modulating the expression of *ABP* genes (Shen et al., 2016). AaJAZ proteins act as the main repressors of jasmonate signaling and are degraded by JA - Ile through their ZIM and Jas domains. The importance of *AaJAZ8* in the jasmonate signaling pathway of *A. annua* has been demonstrated by earlier studies showing its ability to repress several transcription factors. Y2H assays confirmed in the present work that AaJAZ8 physically binds to two novel MYCs, AaMYC24 and AaMYC26, which are specifically induced by MeJA in OL. These results suggest that AaJAZ8 - AaMYC2 module widely controls artemisinin biosynthetic genes. By contrast, AaJAZ8 - AaMYC24/26 module validated by Y2H allows tissue - selective regulation of jasmonate responses based on OL specific interactions, which agrees with OL specific transcriptomic changes seen in this study. Because Y2H is a preliminary method and the low interface predicted template modeling (ipTM) score from AlphaFold3, these candidate interactions should be confirmed by other methods (e.g., bimolecular fluorescence complementation (BiFC) or co - immunoprecipitation (co - IP)). As indicated in the Y2H result (**Figure 4b**), AaJAZ8 is able to form a homodimer but AlphaFold3 estimates an ipTM of just 0.24 of this homodimer (**Supplementary Table 9**). Since an ipTM less than 0.6 is considered low confidence, the prediction value of 0.24 effectively means that AlphaFold3 could not produce a meaningful model of this interaction.

JAZ proteins are known to possess intrinsically disordered regions, and their homodimerization may involve dynamic or transient interactions that are challenging for static structure prediction algorithms to capture. Therefore, the Y2H result is considered experimentally valid, and the discrepancy is attributed to the limitations of AlphaFold3 in modeling this specific homodimer. The low ipTM score should not be regarded as evidence against homodimer formation. AaJAZ1, AaJAZ12, and AaJAZ18 are all capable of forming heterodimers with AaJAZ8. However, the expression levels of these three *AaJAZ*s remained unaffected by short - term MeJA treatment, which implies the complexity of AaJAZ heterodimer regulation. Moreover, TFs that exhibited a significant correlation but no direct interaction with AaJAZ8 may exert their functions through other mechanisms. For instance, it is plausible that these TFs participate in the jasmonic acid response by binding to the promoter regions of AaJAZ8 or other related genes. Significantly, this study revealed that AaJAZ8 functions within an extensive interaction network in the jasmonate signaling network of *A. annua* via Y2H and transcriptomic analyses. Because the genetic transformation has low efficiency and long cycle, the transgenic validation was not included in this study. Even though (Yuan et al., 2025) were able to report successful transgenic experiments of *AaMYC3* in *A. annua*, they also noted that these measures are still time-consuming and expensive. Moreover, during the period when this research was carried out our laboratory did not have a uniform and stable method of genetic transformation in *A. annua*. We thus used other approaches like Y2H systems and qPCR to back up our findings. Nevertheless, this work offers a molecular basis of the subsequent investigation. Future studies must confirm the role of *AaJAZ8* by overexpressing/CRISPR knockout or VIGS, and come up with a good transformation system to confirm the results of this article.

## 5 Conclusion

In this study, we conducted a systematic investigation of *AaJAZ*s in the two haplotype genomes of *A. annua* LQ-9. RNA-Seq analysis revealed distinct response patterns to MeJA across different leaves, with 10 *AaJAZ*s showing significant responses to MeJA in OL. Further interaction network analysis and Y2H assays led to the identification of two novel AaMYC proteins that interact with AaJAZ8. Additionally, AaJAZ8 was able to form homodimers and heterodimers with 10 other AaJAZ proteins. These findings further support that AaJAZ8 functions within an extensive interaction network involved in MeJA responses and hold significant implications for stress response research in *A. annua.*

## Data Availability

The *AaJAZ* sequences and transcriptome data generated from this study are available at GPGD (http://www.gpgenome.com/species/92).

## Supplementary material

Supplementary Table 1 Primers for validation of alternative splicing and genetic variations in allelic *AaJAZ* genes.

Supplementary Table 2 Statistics of clean data of RNA-Seq of MeJA treatment.

Supplementary Table 3 Primers for qPCR detection.

Supplementary Table 4 Primers with homology arms for vector construction.

Supplementary Table 5 *AaJAZ* genes and domains in the two haplotype genomes of *A. annua* LQ-9.

Supplementary Table 6 Characteristics of *AaJAZ*s.

Supplementary Table 7 Statistics of genetic differences between allelic *AaJAZ*s.

Supplementary Table 8 *AaMYC*s in genome of LQ-9 h0.

Supplementary Table 9 Prediction of protein-protein interaction.

Supplementary Table 10 Interactions between AaJAZ proteins and transcription factors.

Supplementary Figure 1 Analysis of vector double digestion and target gene amplification by agarose gel electrophoresis.

Supplementary Figure 2 Phylogenetic tree of AaJAZ proteins and JAZs from *Arabidopsis*.

Supplementary Figure 3 Validation of SNPs between *AaJAZ4* allelic genes.

Supplementary Figure 4 Alternative splicing of *AaJAZ7*, *AaJAZ14* and *AaJAZ15*.

Supplementary Figure 5 DEGs in different leaves treated with MeJA.

Supplementary Figure 6 GO enrichment of up-regulated **(A)** and down-regulated **(B)** genes in two or more leaf types.

Supplementary Figure 7 qPCR validation of ten differentially expressed *AaJAZ*s.

Supplementary Figure 8 Expression profile of the ABP and SCF complex genes in *A. annua* with MeJA treatment.

Supplementary Figure 9 Co-expression analysis of DEGs and GO enrichment of genes in brown module.

Supplementary Figure 10 Expression profile of 12 related genes obtained in the co-expression analysis in brown module.
