# Supplementary material for: AaJAZ8 forms an extensive interaction network with AaJAZ proteins and two novel AaMYC transcription factors in Artemisia annua
Source: Front Plant Sci. 2026 Jun 11;17:1855917. doi: 10.3389/fpls.2026.1855917 (PMC13294869; doi:10.3389/fpls.2026.1855917)
Supplement: Supplementary Table 1 — Primers for validation of alternative splicing and genetic variations in allelic AaJAZ genes. [file DataSheet2.docx]

Table S1 Primers for validation of alternative splicing and genetic variations in allelic *AaJAZ*s.

| Primer name | Primer sequences (5’-3’) | Amplification length (bp) | Template and application |
| --- | --- | --- | --- |
| AaJAZ8_AS | AATCTCGTTCCCTCCTCC | 988/896 | cDNA, validation of alternative splicing |
|  | TGGCACATATTTCACCGT |  |  |
| AaJAZ8_GV | AAAATCTCGTTCCCTCCTCCCA | 1018 | cDNA, Validation of genetic variation |
|  | GCAGATCCCAAAATTCAAATCC |  |  |
| AaJAZ4_GV | GATTCCGGTGACACCAAG | 675 | cDNA, Validation of genetic variation |
|  | CCAACATATCAACGAAAAGG |  |  |

Table S2 Statistics of clean data of RNA-Seq of MeJA treatment.

| Sample | Read number | Total data | GC content | Q20 | Q30 |
| --- | --- | --- | --- | --- | --- |
| MeJA0_TL_1 | 59589080 | 8913770067 | 43.7709 | 99.4361 | 98.0443 |
| MeJA0_TL_2 | 57053074 | 8532023201 | 43.7149 | 99.4679 | 98.1453 |
| MeJA0_TL_3 | 58146102 | 8691256411 | 43.636 | 99.4797 | 98.1793 |
| MeJA0_ML_1 | 54432016 | 8140691727 | 43.4025 | 99.3728 | 97.8593 |
| MeJA0_ML_2 | 55147556 | 8249078930 | 43.2864 | 99.398 | 97.9351 |
| MeJA0_ML_3 | 56318528 | 8422896967 | 43.2866 | 99.4385 | 98.0729 |
| MeJA0_OL_1 | 60005140 | 8972404214 | 43.2423 | 99.4394 | 98.0799 |
| MeJA0_OL_2 | 58863168 | 8803977181 | 43.0992 | 99.4859 | 98.1704 |
| MeJA0_OL_3 | 60503358 | 9050575916 | 43.1169 | 99.4199 | 97.9585 |
| MeJA100_TL_1 | 61206498 | 9154345454 | 43.6881 | 99.4561 | 98.0804 |
| MeJA100_TL_2 | 62889026 | 9394676952 | 43.8546 | 99.4637 | 98.1581 |
| MeJA100_TL_3 | 55257110 | 8260773756 | 43.7373 | 99.427 | 98.0422 |
| MeJA100_ML_1 | 71618624 | 10708028241 | 44.0308 | 99.4863 | 98.2078 |
| MeJA100_ML_2 | 54047332 | 8084759546 | 43.5712 | 99.3688 | 97.84 |
| MeJA100_ML_3 | 67525938 | 10096395434 | 43.8014 | 99.4247 | 98.0297 |
| MeJA100_OL_1 | 53261620 | 7968668361 | 43.1969 | 99.4457 | 98.0552 |
| MeJA100_OL_2 | 64854206 | 9698615728 | 43.4126 | 99.386 | 97.9136 |
| MeJA100_OL_3 | 106545168 | 15932978502 | 43.3292 | 99.479 | 98.2121 |

“MeJA100” represents treatment with 100 µmol/L MeJA, and “MeJA0” represents the control.

Table S3 Primers for qPCR detection.

| Gene | Forward primer (5’-3’) | Reverse primer (5’-3’) | Amplification length (bp) |
| --- | --- | --- | --- |
| *AaJAZ1* | TTGGATCACATCTTATAGGGG | TAACAGGAACGGCTCGG | 273 |
| *AaJAZ2* | GGCTAAAACCTCGCAAA | TAGGCAAATCCGAATCC | 209 |
| *AaJAZ3* | TCGCTCAAATGACACTCC | GTACACCTGTTCAGCCAGA | 154 |
| *AaJAZ4* | TACACCAGCATCAGCCC | TTCTCGCCATTGGAAGA | 155 |
| *AaJAZ5* | CCTCTTCCTGAGTATGTTAGTT | GAAGTTGAAGCCTTTTGAAC | 233 |
| *AaJAZ6* | TCTCCATCACCATCCCC | CATGACCCTGCATCTGC | 233 |
| *AaJAZ7* | ATCTCAGGCGATCCAGC | AGGCACCGATTCTTCAG | 163 |
| *AaJAZ8* | TCCAAGGTTGCTTCTGC | CCAAAAGGGTGTGACGA | 248 |
| *AaJAZ9* | CTCAAGGATGAATCTTCTACTC | GAATATAGTCAAAGGAGGACC | 249 |
| *AaJAZ10* | TGTAACTTAACGCTCCGC | CAAACACTAACCATCCCATC | 152 |
| *AaJAZ11* | CCCATTGGTTTATCTCCC | CGCTGCTACGGTTTTGTT | 182 |
| *AaJAZ12* | AAACCACTCCACCTACTCAC | TTCAAGCAACGCCTTAA | 150 |
| *AaJAZ13* | GAAGGGTACTCACACCATCA | TCTGTCCACTTTCAGCAAC | 173 |
| *AaJAZ14* | ATCTCCTACTGGTGTTCTGG | AAGGCTTCCTGTGCTCC | 262 |
| *AaJAZ15* | TGTCACTAAGGTGGTTGCT | CGGATTATTGCGTTTACTC | 238 |
| *AaJAZ16* | CGAGAACCCCAGCAGAT | TCAAGAAAGCGAGCCAA | 149 |
| *AaJAZ17* | CTATGGTCCGAATCTAGTACAT | GCTCTTTTGGTGACGAAT | 176 |
| *AaJAZ18* | ATGGTCCGAATCTAGTACATG | GCTCCTTTGGTGACGAAT | 174 |

Table S4 Primers for vector construction.

| Primer name | Primer sequence (5’-3’) |
| --- | --- |
| PGB-AaJAZ8-F | tcagaggaggacctgcatatgATGGAGAGGGATTTTATGGGTTT |
| PGB-AaJAZ8-R | ccgctgcaggtcgacggatccTCACTTGCTTGCAACAGCGA |
| PGA- AaMYC26-F | gtaccagattacgctcatatgATGAGTATTGAAAGTTTTAATGATGAGGA |
| PGA- AaMYC26-R | atgcccacccgggtggaattcTTATGGTAATGAGTTTAAAGAGGAGCTT |
| PGA-AaJAZ1-F | agattacgctcatatggccatggaggccATGTCATCACTGTTTTTGTTAAACAACA |
| PGA-AaJAZ1-R | cagctcgagctcgatggatccTCACAATTTCTCTTCAGTACATGGC |
| PGA-AaJAZ2-F | agattacgctcatatggccatggaggccATGTCGGCTGCCCAACGT |
| PGA-AaJAZ2-R | cagctcgagctcgatggatccAATCATATTAAGGTCGAAACTAAGCTCG |
| PGA-AaJAZ3-F | agattacgctcatatggccatggaggccATGTACGGAGAGACCGAGTTTGTT |
| PGA-AaJAZ3-R | cagctcgagctcgatggatccTCAGTTACCAAACCCGAGCTCA |
| PGA-AaJAZ4-F | gtaccagattacgctcatatgATGTCTGAGACTGTGGATTCCGG |
| PGA-AaJAZ4-R | atgcccacccgggtggaattcTTATTGGGCACCAGAAGATTCAG |
| PGA-AaJAZ5-F | gtaccagattacgctcatatgATGTCGATGGCTAGAAACTTTTTTAA |
| PGA-AaJAZ5-R | atgcccacccgggtggaattcCTATAAGTTGAGATCAAATTTATAATTCAAAGA |
| PGA-AaJAZ6-F | gtaccagattacgctcatatgATGTCAGAACCAGATATGAATCACACC |
| PGA-AaJAZ6-R | atgcccacccgggtggaattcTCACCTTTCAACGCCCACA |
| PGA-AaJAZ7-F | agattacgctcatatggccatggaggccATGGAAGAACCACCATCTGCC |
| PGA-AaJAZ7-R | cagctcgagctcgatggatccTTAGCTAAACCGGTTAGGGGTG |
| PGA-AaJAZ8-F | agattacgctcatatggccatggaggccATGGAGAGGGATTTTATGGGTTT |
| PGA-AaJAZ8-R | cagctcgagctcgatggatccTCACTTGCTTGCAACAGCGA |
| PGA-AaJAZ9-F | agattacgctcatatggccatggaggccATGTTGAGATCACCCTCGGTTG |
| PGA-AaJAZ9-R | cagctcgagctcgatggatccTCAATGCAAAGACAAGTCTCCTTG |
| PGA-AaJAZ10-F | agattacgctcatatggccatggaggccATGAGGAGGAATTGTAACTTAACGC |
| PGA-AaJAZ10-R | cagctcgagctcgatggatccCTAGCGGTGGTAGGGAGAAGTAGC |
| PGA-AaJAZ11-F | agattacgctcatatggccatggaggccATGTTTCTCACACACATGTACATGCA |
| PGA-AaJAZ11-R | cagctcgagctcgatggatccCTAGTGATGGTAAGGAGATGTTGCTT |
| PGA-AaJAZ12-F | agattacgctcatatggccatggaggccATGACTGCCACGTCATCACCA |
| PGA-AaJAZ12-R | cagctcgagctcgatggatccTAGCTGTAGCGGTTAGGGGTATTA |
| PGA-AaJAZ13-F | agattacgctcatatggccatggaggccATGGGTTCATCAGAAATTGTGGA |
| PGA-AaJAZ13-R | cagctcgagctcgatggatccTTATTGGACATGAGATTGTGCACC |
| PGA-AaJAZ14-F | agattacgctcatatggccatggaggccATGGAACGAGATTTTATGGGTTTG |
| PGA-AaJAZ14-F | cagctcgagctcgatggatccCTAATGCTCGCCTGCTGCTAC |
| PGA-AaJAZ15-F | gtaccagattacgctcatatgATGGCTGATGTGTTGTTGTTGTTAA |
| PGA-AaJAZ15-R | atgcccacccgggtggaattcTCACTGTTTTTCATCACTCTTTTGTTC |
| PGA-AaJAZ16-F | agattacgctcatatggccatggaggccATGGAAAGGGATTTCATGGGTT |
| PGA-AaJAZ16-R | cagctcgagctcgatggatccTCACTTTGTATCATCTTTGCTCGG |
| PGA-AaJAZ17-F | agattacgctcatatggccatggaggccATGTCATCAGCTAAAGAATTCGGC |
| PGA-AaJAZ17-R | cagctcgagctcgatggatccCTATAAATTTAGATCGAACTTATGCTCTTTT |
| PGA-AaJAZ18-F | agattacgctcatatggccatggaggccATGTCATCGGCTAAACAATTCGG |
| PGA-AaJAZ18-R | cagctcgagctcgatggatccCTATAAATTTAGATCGAACTTATGCTCCT |
| PGA-AaMYC24-F | agattacgctcatatggccatggaggccATGAAAAGCGAAGCTAGTATGGTTAT |
| PGA- AaMYC24-R | cagctcgagctcgatggatccTCACGAAGAGCTGGTTTCTCG |
| PGA-g00055751-F | agattacgctcatatggccatggaggccATGGAGCACCAAGAAAATGTTGT |
| PGA-g00055751-R | cagctcgagctcgatggatccCTAATGAACAACCTCTTCAGAAATCG |
| PGA-g00317131-F | agattacgctcatatggccatggaggccATGGAAGACATTGGTGATGAATACA |
| PGA-g00317131-R | cagctcgagctcgatggatccCTAGGTGCTCATTGGACTCGGA |
| PGA-g00317101-F | agattacgctcatatggccatggaggccATGGAAGACATTGGTGATGAATACA |
| PGA-g00317101-R | cagctcgagctcgatggatccCTAGGTGCTCATTGGACTTGGAG |
| PGA-g01026541-F | agattacgctcatatggccatggaggccATGGGAAGAACACCATGCTGTT |
| PGA-g01026541-R | cagctcgagctcgatggatccTTAATTAACACCATCTACACCTTCGTAA |
| PGB-AaJAZ4-F | tcagaggaggacctgcatatgATGTCTGAGACTGTGGATTCCGG |
| PGB-AaJAZ4-R | tcgacggatccccgggaattcTTATTGGGCACCAGAAGATTCAG |
| PGB-AaJAZ10-F | ggaggacctgcatatggccatggaggccATGAGGAGGAATTGTAACTTAACGC |
| PGB-AaJAZ10-R | ccgctgcaggtcgacggatccCTAGCGGTGGTAGGGAGAAGTAGC |
| PGB-AaJAZ13-F | ggaggacctgcatatggccatggaggccATGGGTTCATCAGAAATTGTGGA |
| PGB-AaJAZ13-R | ccgctgcaggtcgacggatccTTATTGGACATGAGATTGTGCACC |
| PGB-AaJAZ16-F | ggaggacctgcatatggccatggaggccATGGAAAGGGATTTCATGGGTT |
| PGB-AaJAZ16-R | ccgctgcaggtcgacggatccTCACTTTGTATCATCTTTGCTCGG |
| PGB-AaJAZ17-F | ggaggacctgcatatggccatggaggccATGTCATCAGCTAAAGAATTCGGC |
| PGB-AaJAZ17-R | ccgctgcaggtcgacggatccCTATAAATTTAGATCGAACTTATGCTCTTTT |

Lowercase letters represent homologous arm sequences, and red indicates the enzymatic cleavage sites.

Table S5 *AaJAZ*s and domains in the two haplotype genomes of *A. annua* LQ-9.

| LQ-9 h0 alleles | LQ-9 h1 alleles | Name | PF06200 TIFY_domain | PF09425 Jas_motif (CCT_2) | PF06203 CCT | PF00320 GATA |
| --- | --- | --- | --- | --- | --- | --- |
| chr1g00032841 | chr1g00031151 | *AaJAZ1* | 1/1 | 0/0 | 0/0 | 0/0 |
| chr1g00093051 | chr1g00090781 | *AaJAZ2* | 1/1 | 1/1 | 0/0 | 0/0 |
| chr1g00191741 | chr1g00167851 | *AaJAZ3* | 1/1 | 0/0 | 1/1 | 1/1 |
| chr2g00302791 | chr2g00443911 | *AaJAZ4* | 1/1 | 1/1 | 0/0 | 0/0 |
| chr2g00328291 | chr2g00470631 | *AaJAZ5* | 1/1 | 1/1 | 0/0 | 0/0 |
| chr2g00360331 | chr2g00500201 | *AaJAZ6* | 1/1 | 0/0 | 1/1 | 1/1 |
| chr2g00407501 | chr2g00541981 | *AaJAZ7* | 1/1 | 1/1 | 0/0 | 0/0 |
| chr3g00516701 | chr3g00641251 | *AaJAZ8* | 1/1 | 1/1 | 0/0 | 0/0 |
| chr3g00536111 | chr3g00659651 | *AaJAZ9* | 1/1 | 1/1 | 0/0 | 0/0 |
| chr4g00689071 | chr4g00803231 | *AaJAZ10* | 1/1 | 1/1 | 0/0 | 0/0 |
| chr4g00689081 | chr4g00803241 | *AaJAZ11* | 1/1 | 1/1 | 0/0 | 0/0 |
| chr4g00710761 | chr4g00823191 | *AaJAZ12* | 1/1 | 1/1 | 0/0 | 0/0 |
| chr4g00725711 | chr4g00868411 | *AaJAZ13* | 1/1 | 1/1 | 0/0 | 0/0 |
| chr5g00913181 | chr5g00334121 | *AaJAZ14* | 1/1 | 1/1 | 0/0 | 0/0 |
| chr6g00988241 | chr6g00929251 | *AaJAZ15* | 1/1 | 0/0 | 0/0 | 0/0 |
| chr8g01283201 | chr8g01065131 | *AaJAZ16* | 1/1 | 1/1 | 0/0 | 0/0 |
| chr9g01350381 | chr9g01242621 | *AaJAZ17* | 1/1 | 1/1 | 0/0 | 0/0 |
| chr9g01350401 | - | *AaJAZ18* | 1/- | 1/- | 0/- | 0/- |

“-” represent absence; the data on the left of the “/” represents LQ-9 h0, while that on the right represents LQ-9 h1.

Table S6 Information of *AaJAZ*s.

| Gene | Amino acids | Isoelectric Point | Molecular weight | Gravity | Protein localization | Protein identity (%) | Exon number |
| --- | --- | --- | --- | --- | --- | --- | --- |
| *AaJAZ1* | 360/360 | 7.13/7.76 | 38.18/38.14 | -0.461/-0.480 | Nucleus/Nucleus | 98.61 | 6/6 |
| *AaJAZ2* | 237/250 | 8.4/8.84 | 26.06/27.43 | -0.668/-0.688 | Nucleus/Nucleus | 87.20 | 2/2 |
| *AaJAZ3* | 269/269 | 5.99/5.99 | 29.42/29.42 | -0.710/-0.710 | Nucleus/Nucleus | 100.00 | 7/7 |
| *AaJAZ4* | 154/154 | 5.39/5.39 | 16.95/16.97 | -0.578/-0.600 | Nucleus/Nucleus | 99.35 | 4/4 |
| *AaJAZ5* | 221/221 | 5.99/5.99 | 24.63/24.62 | -0.547/-0.561 | Nucleus/Nucleus | 99.10 | 2/2 |
| *AaJAZ6* | 358/362 | 5.66/5.71 | 39.13/39.56 | -0.890/-0.875 | Nucleus/Nucleus | 96.41 | 7/7 |
| *AaJAZ7* | 305/305 | 8.78/8.78 | 33.88/33.86 | -0.843/-0.858 | Nucleus/Nucleus | 99.67 | 8/8 |
| *AaJAZ8* | 255/255 | 9.14/9.14 | 27.69/27.68 | -0.369/-0.371 | Nucleus/Nucleus | 99.61 | 8/8 |
| *AaJAZ9* | 186/186 | 9.54/9.52 | 20.36/20.39 | -0.511/-0.533 | Nucleus/Nucleus | 98.93 | 4/4 |
| *AaJAZ10* | 130/130 | 9.1/9.46 | 14.86/14.88 | -0.815/-0.803 | Nucleus/Nucleus | 98.46 | 3/3 |
| *AaJAZ11* | 194/138 | 9.33/9.26 | 22.74/15.75 | -0.461/-0.773 | Nucleus/Nucleus | 100.00 | 3/3 |
| *AaJAZ12* | 288/292 | 8.19/8.5 | 31.61/31.99 | -0.660/-0.660 | Nucleus/Nucleus | 97.95 | 8/8 |
| *AaJAZ13* | 178/178 | 9.27/9.27 | 19.81/19.78 | -0.562/-0.539 | Nucleus/Nucleus | 98.88 | 4/4 |
| *AaJAZ14* | 318/318 | 8.95/9.08 | 33.50/33.45 | -0.316/-0.325 | Nucleus/Nucleus | 97.48 | 9/9 |
| *AaJAZ15* | 377/375 | 8.89/8.89 | 39.35/38.93 | -0.598/-0.593 | Nucleus/Nucleus | 97.11 | 7/7 |
| *AaJAZ16* | 189/187 | 9.19/9.19 | 20.48/20.26 | -0.698/-0.671 | Nucleus/Nucleus | 97.35 | 6/6 |
| *AaJAZ17* | 191/191 | 9.08/9.25 | 21.29/21.32 | -0.541/-0.502 | Nucleus/Nucleus | 94.24 | 2/2 |
| *AaJAZ18* | 380/- | 9.64/- | 43.20/- | -0.499/- | Nucleus/- | - | 2/- |

“-” represent absence; the data on the left of the “/” represents LQ-9 h0, while that on the right represents LQ-9 h1.

Table S7 Statistics of genetic differences between allelic *AaJAZ*s.

|  | Intron | | | 5'+3' UTR | | | CDS | | | | | | | | |
| --- | --- | --- | --- | --- | --- | --- | --- | --- | --- | --- | --- | --- | --- | --- | --- |
|  | SNP | In | Del | SNP | In | Del | SNP | | | Multiple SNP | | | In (keep the reading frame) | Deletion (keep the reading frame) | In/del (alter the reading frame) |
|  |  |  |  |  |  |  | Syn | Mis | Non | Syn | Mis | Non |  |  |  |
| *AaJAZ1* | 9 | 3 | 0 | 6 | 0 | 3 | 4 | 5 | 0 | 0 | 0 | 0 | 0 | 0 | 0 |
| *AaJAZ2* | 20 | 2 | 8 | 23 | 4 | 4 | 4 | 15 | 0 | 0 | 4 | 0 | 5 | 0 | 0 |
| *AaJAZ3* | 16 | 3 | 7 | 0 | 0 | 0 | 0 | 0 | 0 | 0 | 0 | 0 | 0 | 0 | 0 |
| *AaJAZ4* | 4 | 2 | 4 | 6 | 1 | 1 | 3 | 1 | 0 | 0 | 0 | 0 | 0 | 0 | 0 |
| *AaJAZ5* | 0 | 0 | 0 | 3 | 0 | 0 | 4 | 2 | 0 | 0 | 0 | 0 | 0 | 0 | 0 |
| *AaJAZ6* | 160 | 11 | 16 | 17 | 2 | 1 | 12 | 5 | 0 | 0 | 1 | 0 | 2 | 1 | 0 |
| *AaJAZ7* | 52 | 9 | 10 | 14 | 4 | 4 | 3 | 2 | 0 | 0 | 0 | 0 | 0 | 0 | 1 |
| *AaJAZ8* | 31 | 6 | 5 | 8 | 2 | 0 | 5 | 1 | 0 | 0 | 0 | 0 | 0 | 0 | 0 |
| *AaJAZ9* | 40 | 8 | 5 | 8 | 1 | 3 | 3 | 2 | 0 | 0 | 0 | 0 | 0 | 0 | 0 |
| *AaJAZ10* | 5 | 2 | 0 | 0 | 0 | 1 | 1 | 2 | 0 | 0 | 0 | 0 | 0 | 0 | 0 |
| *AaJAZ11* | 56 | 6 | 6 | 10 | 4 | 0 | 2 | 1 | 0 | 0 | 1 | 0 | 0 | 0 | 3 |
| *AaJAZ12* | 88 | 12 | 9 | 7 | 1 | 2 | 6 | 2 | 0 | 0 | 0 | 0 | 1 | 0 | 0 |
| *AaJAZ13* | 83 | 9 | 15 | 4 | 2 | 0 | 7 | 2 | 0 | 0 | 0 | 0 | 0 | 0 | 0 |
| *AaJAZ14* | 43 | 10 | 6 | 2 | 0 | 1 | 9 | 7 | 0 | 0 | 1 | 0 | 0 | 0 | 0 |
| *AaJAZ15* | 22 | 3 | 4 | 5 | 3 | 2 | 5 | 3 | 0 | 0 | 0 | 0 | 3 | 1 | 0 |
| *AaJAZ16* | 53 | 8 | 9 | 2 | 0 | 0 | 4 | 3 | 0 | 0 | 0 | 0 | 0 | 1 | 0 |
| *AaJAZ17* | 3 | 1 | 0 | 15 | 1 | 3 | 9 | 11 | 0 | 0 | 0 | 0 | 0 | 0 | 0 |

SNP, Single nucleotide polymorphism; In, insertion; Del, deletion; Syn, Synonymous; Mis, missense; Non, Nonsense; Multiple SNP, any two or more of the three bases forming a codon undergo SNP.

Table S8 *AaMYC*s in genome of LQ-9 h0.

| Gene name | Gene number in LQ-9 h0 |
| --- | --- |
| *AaMYC1* | Super-Scaffold_100110g01454181 |
| *AaMYC2* | chr9g01406681 |
| *AaMYC3* | chr9g01380101 |
| *AaMYC4* | chr8g01292121 |
| *AaMYC5* | chr8g01264971 |
| *AaMYC6* | chr7g01138051 |
| *AaMYC7* | chr6g01026361 |
| *AaMYC8* | chr6g01025861a |
| *AaMYC9* | chr6g01025771a |
| *AaMYC10* | chr5g00896821 |
| *AaMYC11* | chr5g00895521 |
| *AaMYC12* | chr5g00879781 |
| *AaMYC13* | chr5g00871451 |
| *AaMYC14* | chr5g00869621 |
| *AaMYC15* | chr5g00866651 |
| *AaMYC16* | chr4g00711781 |
| *AaMYC17* | chr4g00693341 |
| *AaMYC18* | chr4g00670941 |
| *AaMYC19* | chr2g00411561 |
| *AaMYC20* | chr2g00410631 |
| *AaMYC21* | chr2g00404291 |
| *AaMYC22* | chr2g00335571 |
| *AaMYC23* | chr2g00266891 |
| *AaMYC24* | chr2g00266181 |
| *AaMYC25* | chr1g00070091 |
| *AaMYC26* | unctg_3931g01664791 |

Table S9 Prediction of protein-protein interaction.

| Protein 1 | Protein 2 | ipTM | pTM |
| --- | --- | --- | --- |
| AaJAZ8 | AaJAZ8 | 0.24 | 0.24 |
| AaJAZ8 | chr1g00055751 (zinc_finger_ring) | 0.25 | 0.45 |
| AaJAZ8 | AaMYC24 | 0.50 | 0.41 |
| AaJAZ8 | AaJAZ4 | 0.41 | 0.30 |
| AaJAZ8 | chr2g00317101 (bHLH) | 0.20 | 0.24 |
| AaJAZ8 | chr2g00317131 (bHLH) | 0.17 | 0.24 |
| AaJAZ8 | AaJAZ10 | 0.51 | 0.32 |
| AaJAZ8 | AaJAZ13 | 0.34 | 0.29 |
| AaJAZ8 | chr6g01026541 (MYB) | 0.19 | 0.28 |
| AaJAZ8 | AaJAZ16 | 0.33 | 0.28 |
| AaJAZ8 | AaJAZ17 | 0.32 | 0.27 |
| AaJAZ8 | AaMYC26 | 0.43 | 0.38 |

Table S10 Interactions between AaJAZ proteins and transcription factors.

|  | AaMYC24 | AaMYC26 | chr2g00317101  bHLH | chr2g00317131  bHLH | chr6g01026541  MYB | chr1g00055751  Zinc finger |
| --- | --- | --- | --- | --- | --- | --- |
| AaJAZ4 | - | - | - | - | - | - |
| AaJAZ8 | + | + | - | - | - | - |
| AaJAZ10 | + | - | - | - | - | - |
| AaJAZ13 | - | + | - | - | - | - |
| AaJAZ16 | + | - | - | - | - | - |
| AaJAZ17 | + | - | - | - | - | - |

“+” and “-” indicate the presence and absence of an interaction, respectively.


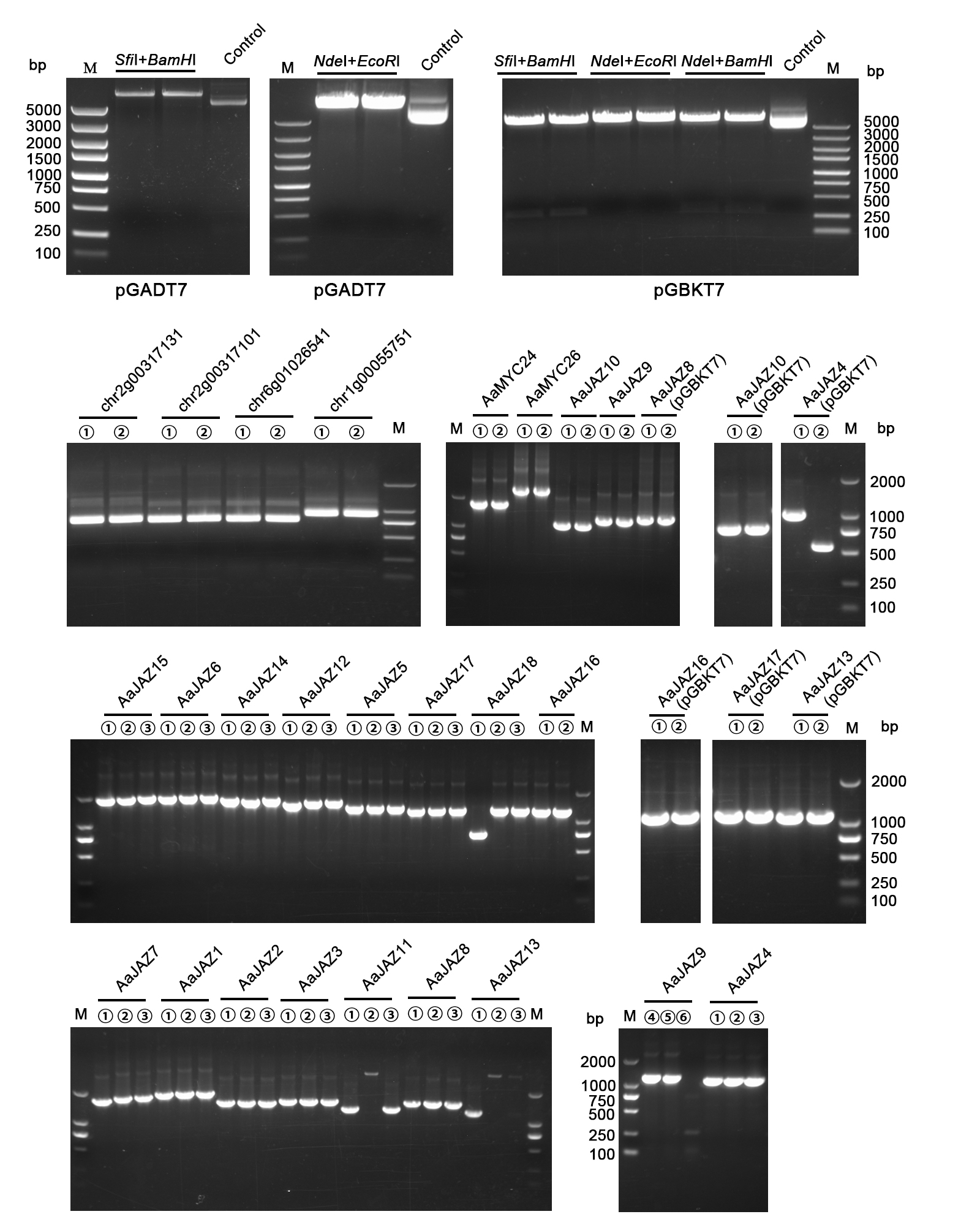


Figure S1 Analysis of vector double digestion and target gene amplification by agarose gel electrophoresis. Amplification with the pGBKT7 universal primers (F: GCTCCAAAGAAAAACCG; R：AAAACCCCTCAAGACCC) yields a product size corresponding to the target gene size plus 561 bp. Amplification with the pGADT7 universal primers (F: TCCGAACCTCATAACAACTC; R: TAGACAAGCCGACAACCT) yields a product size corresponding to the target gene size plus 713 bp.


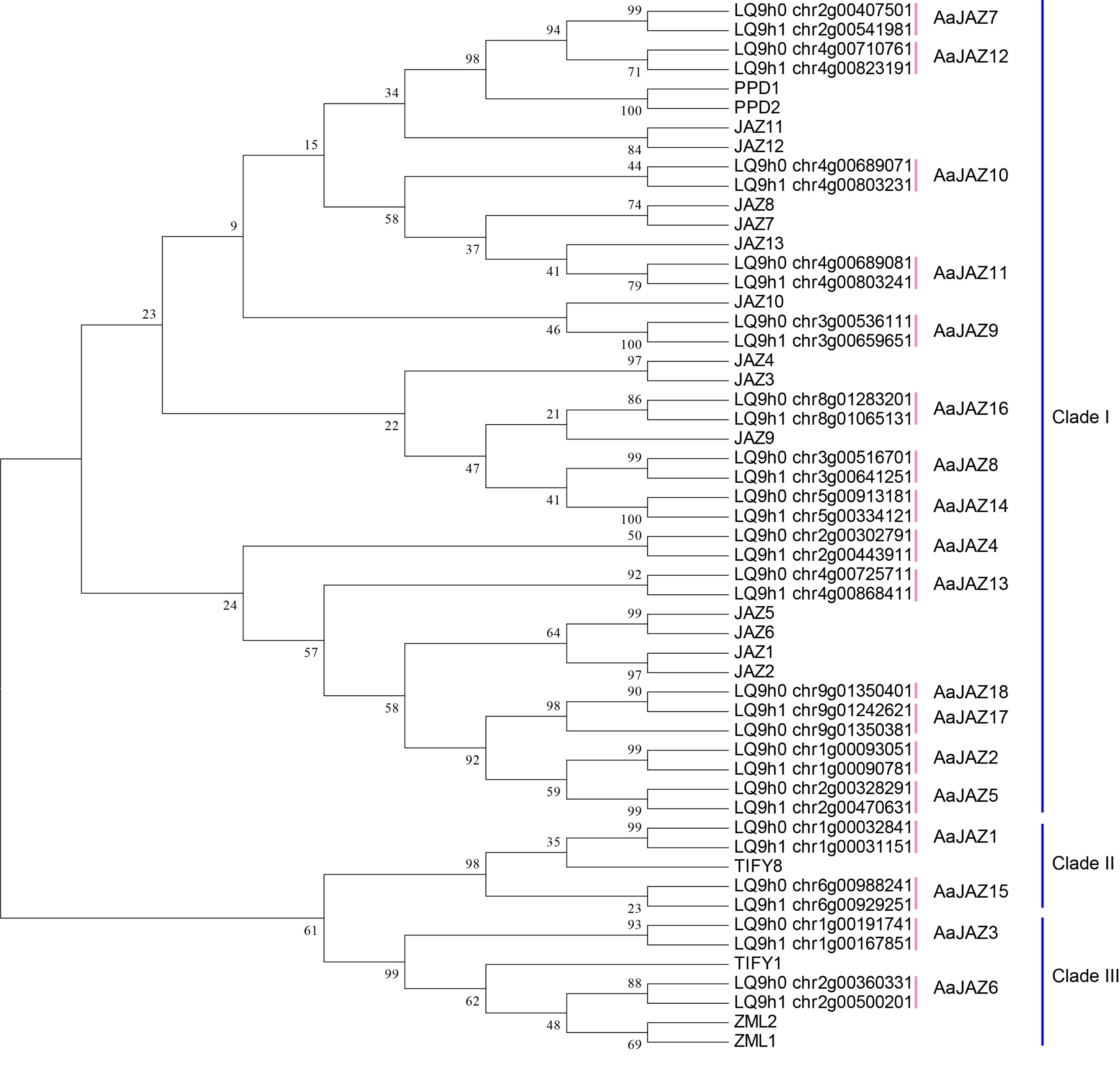


Figure S2 Phylogenetic tree of AaJAZ proteins and JAZs from *Arabidopsis*.


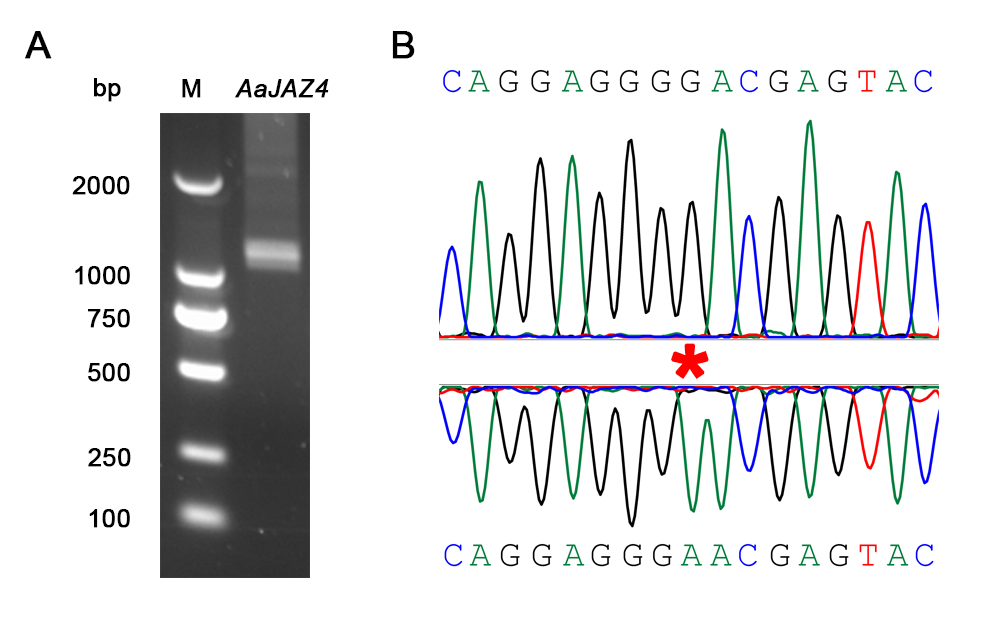


Figure S3 Validation of SNPs between *AaJAZ4* allelic genes.


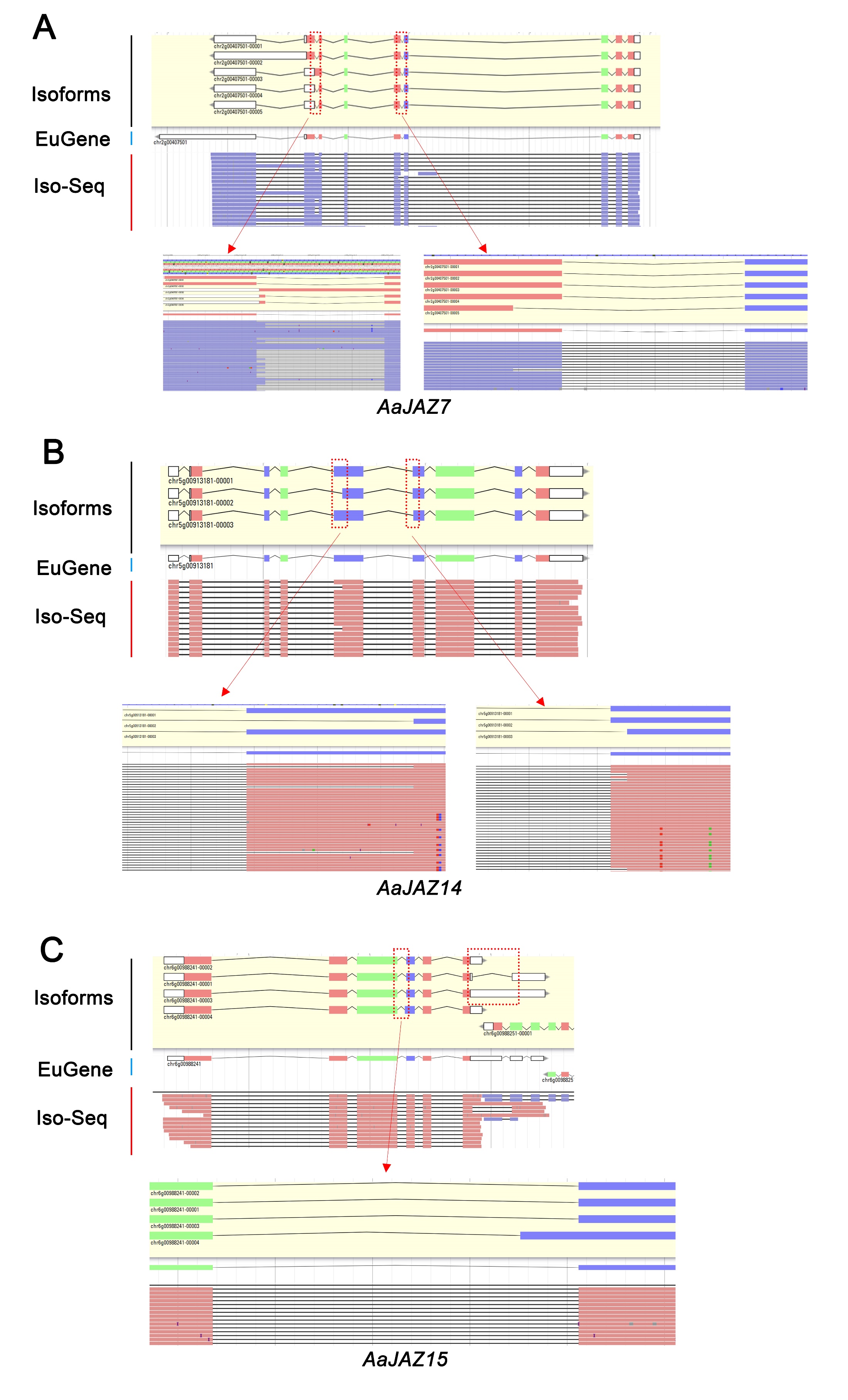


Figure S4 Alternative splicing of *AaJAZ7*, *AaJAZ14* and *AaJAZ15*.


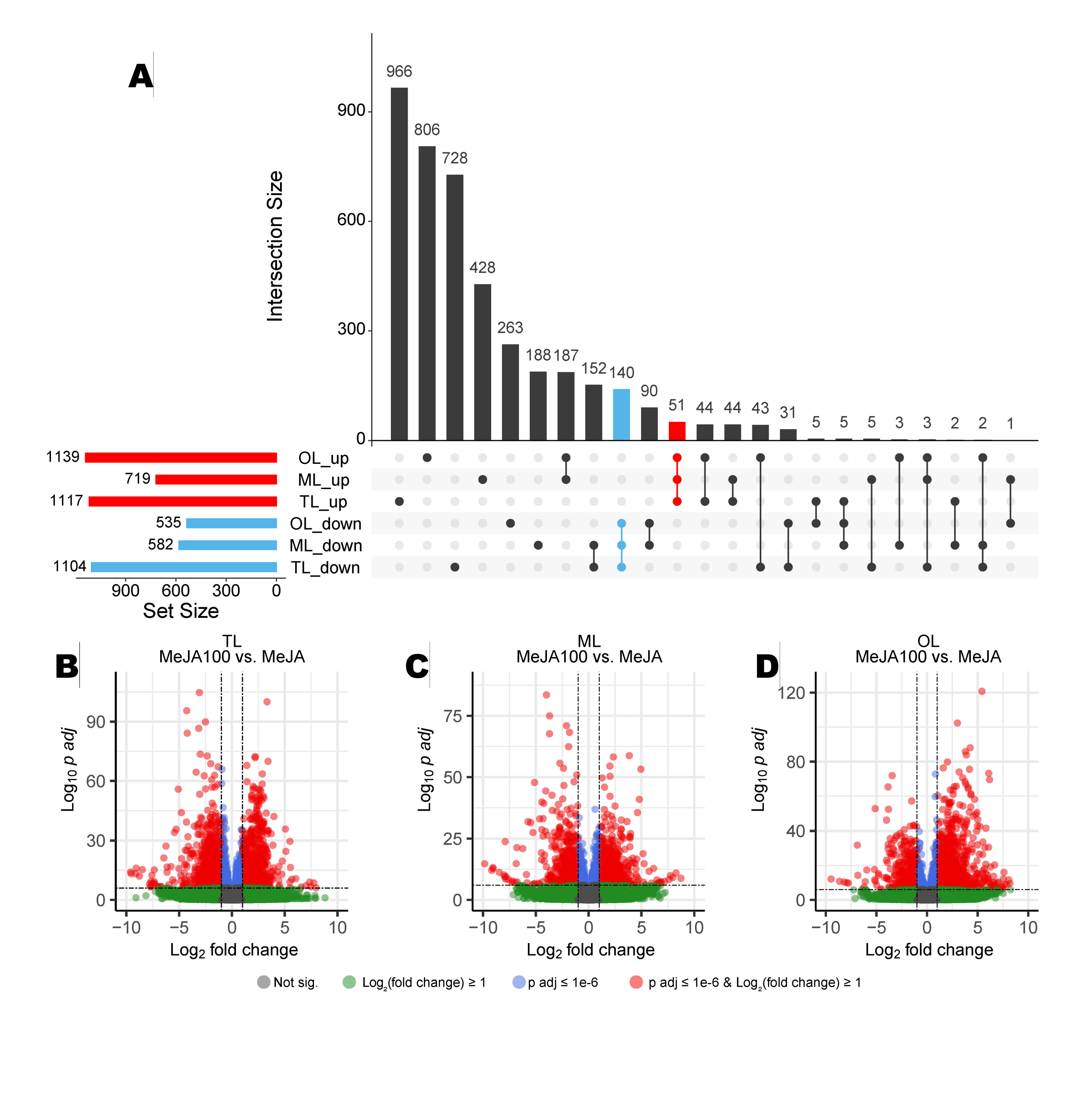


Figure S5 DEGs in different leaves treated with MeJA. (A) UpSet plot illustrating the intersections of up‑regulated and down‑regulated genes across different treatment groups. (B - D) Volcano plots showing differentially expressed genes (DEGs) in each treatment group.


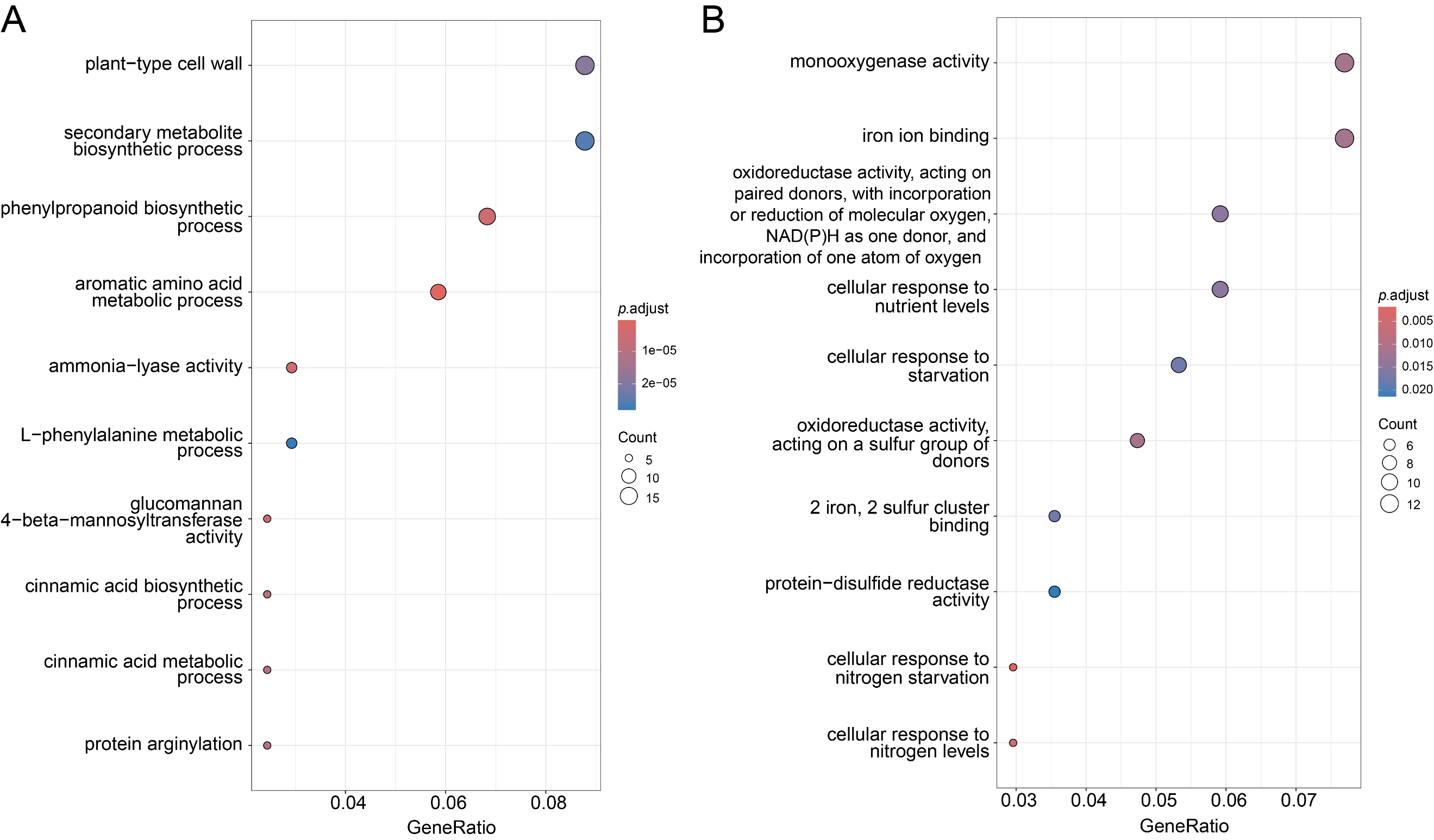


Figure S6 GO enrichment of up-regulated (A) and down-regulated (B) genes in two or more leaf types.


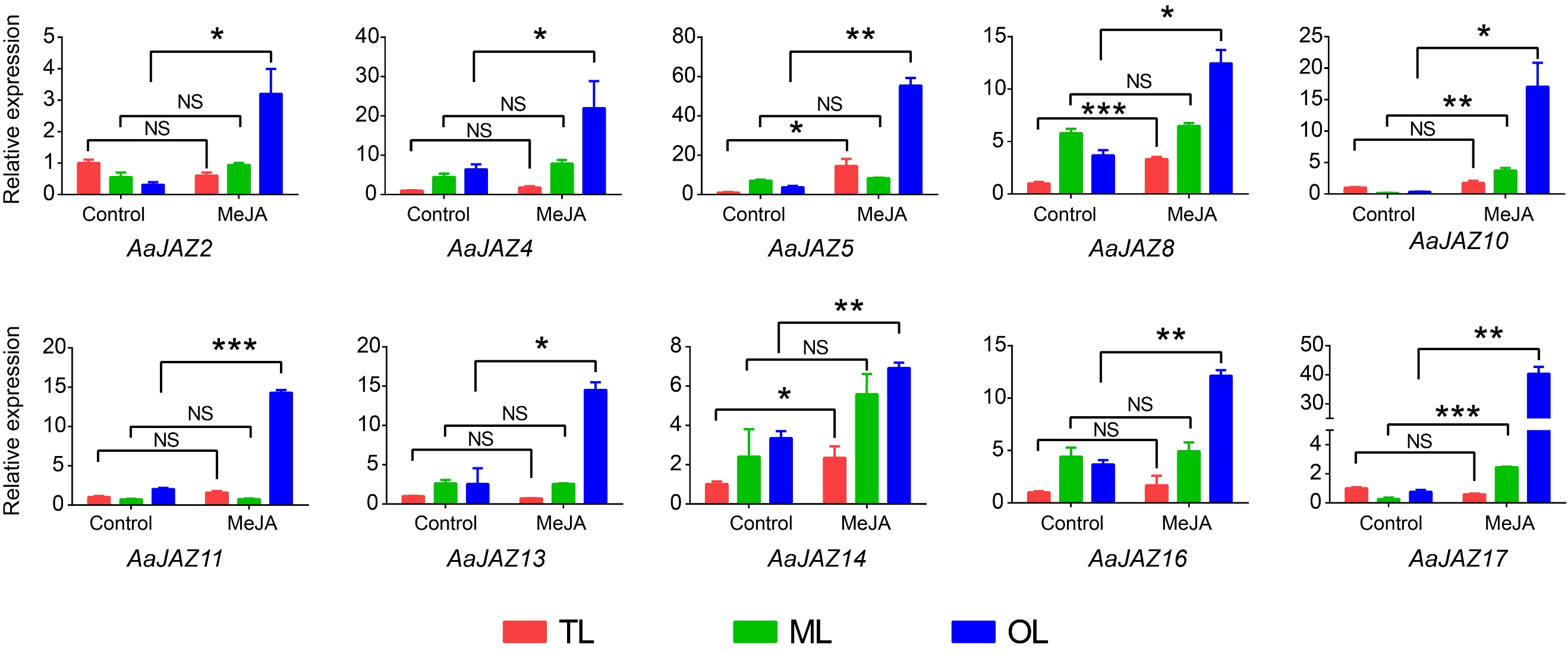


Figure S7 Validation of ten differentially expressed *AaJAZ*s by qPCR. *: foldchange≥2, *p*<0.05; **: foldchange≥2，*p*<0.01; ***: foldchange≥2，*p*<0.001; NS, no significant difference.


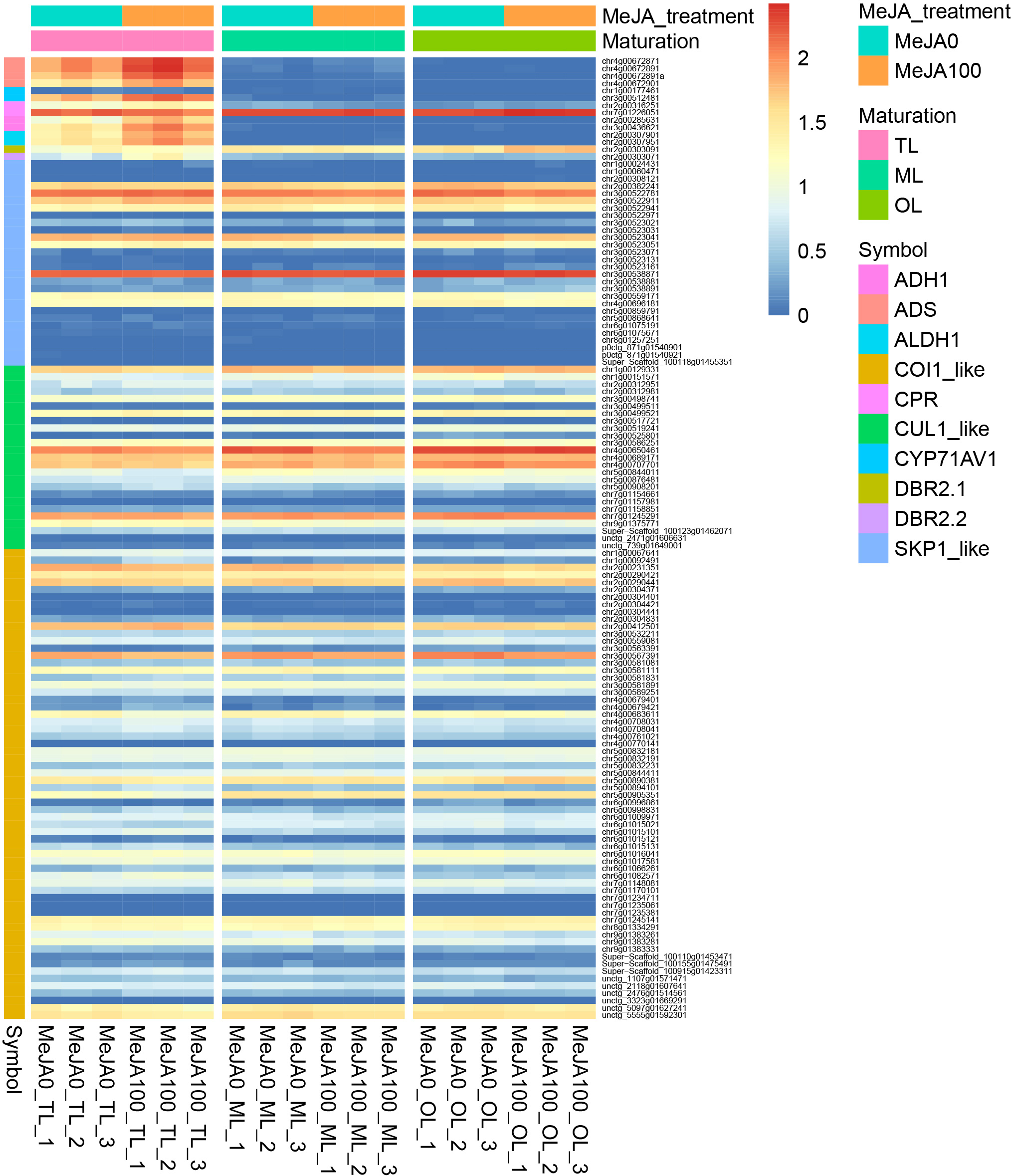


Figure S8 Expression profile of the ABP and SCF complex genes in *A. annua* with MeJA treatment.


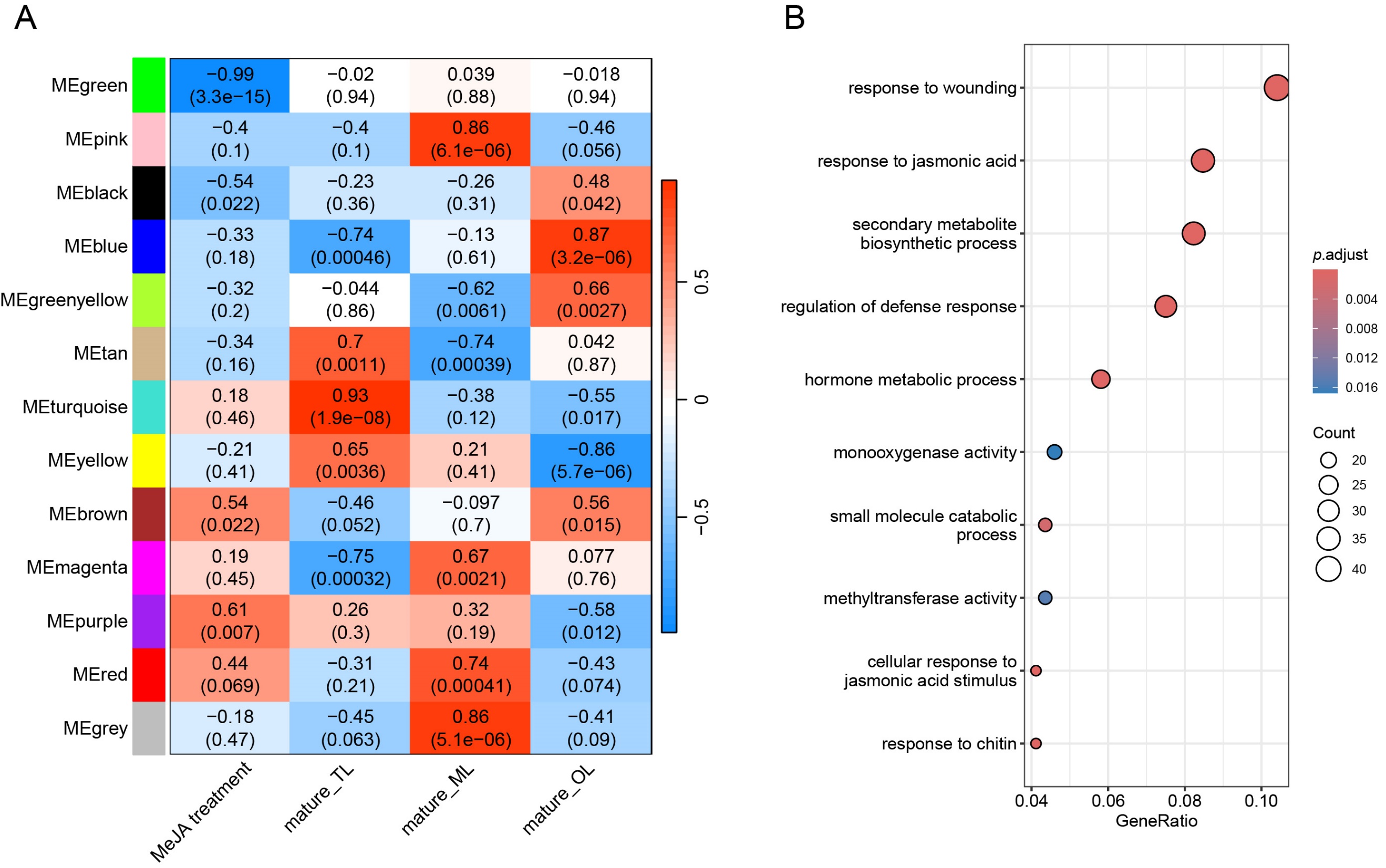


Figure S9 Co-expression analysis of DEGs and GO enrichment of genes in brown module.


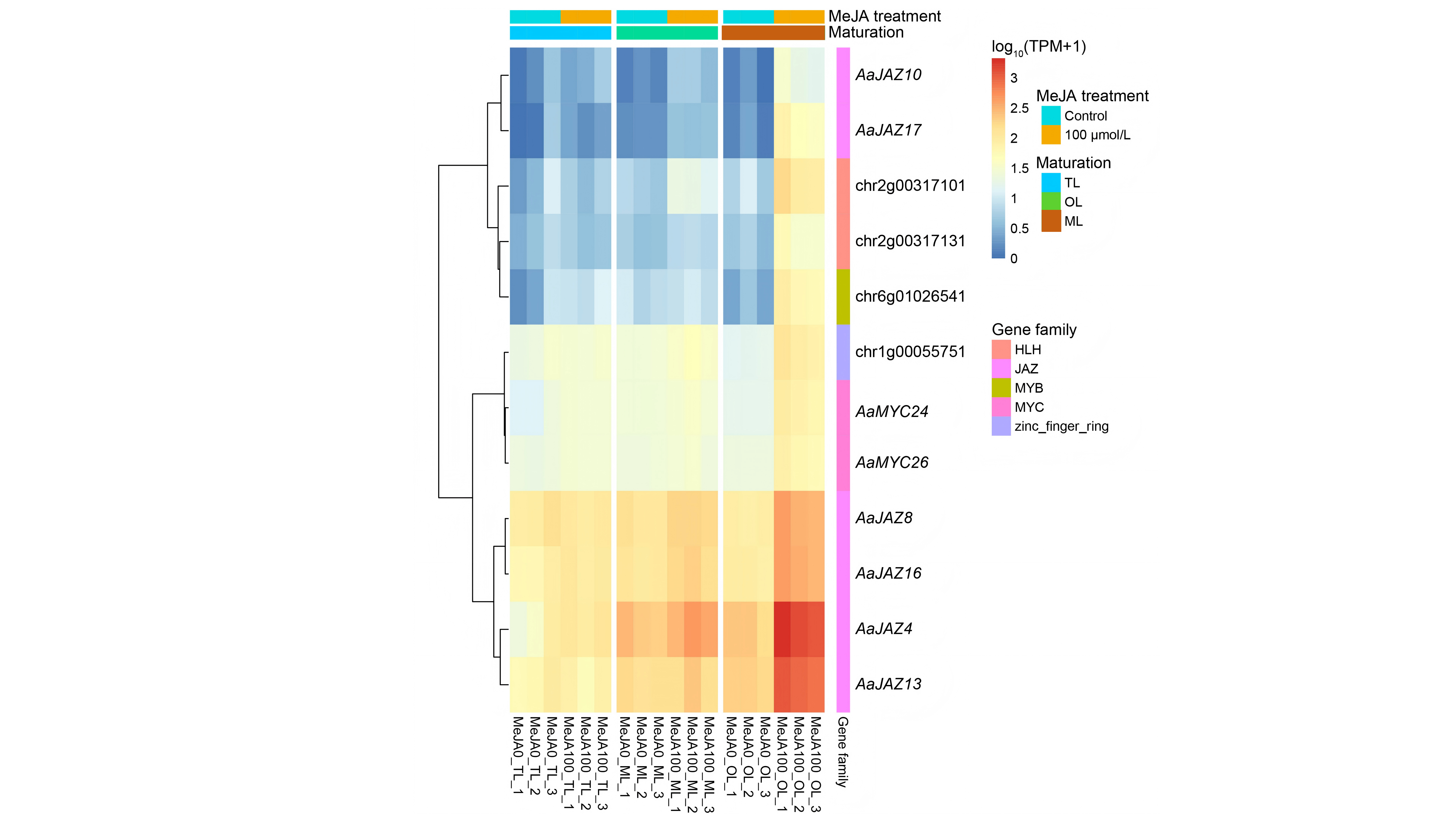


Figure S10 Expression profile of 12 related genes obtained in the co-expression analysis in brown module.


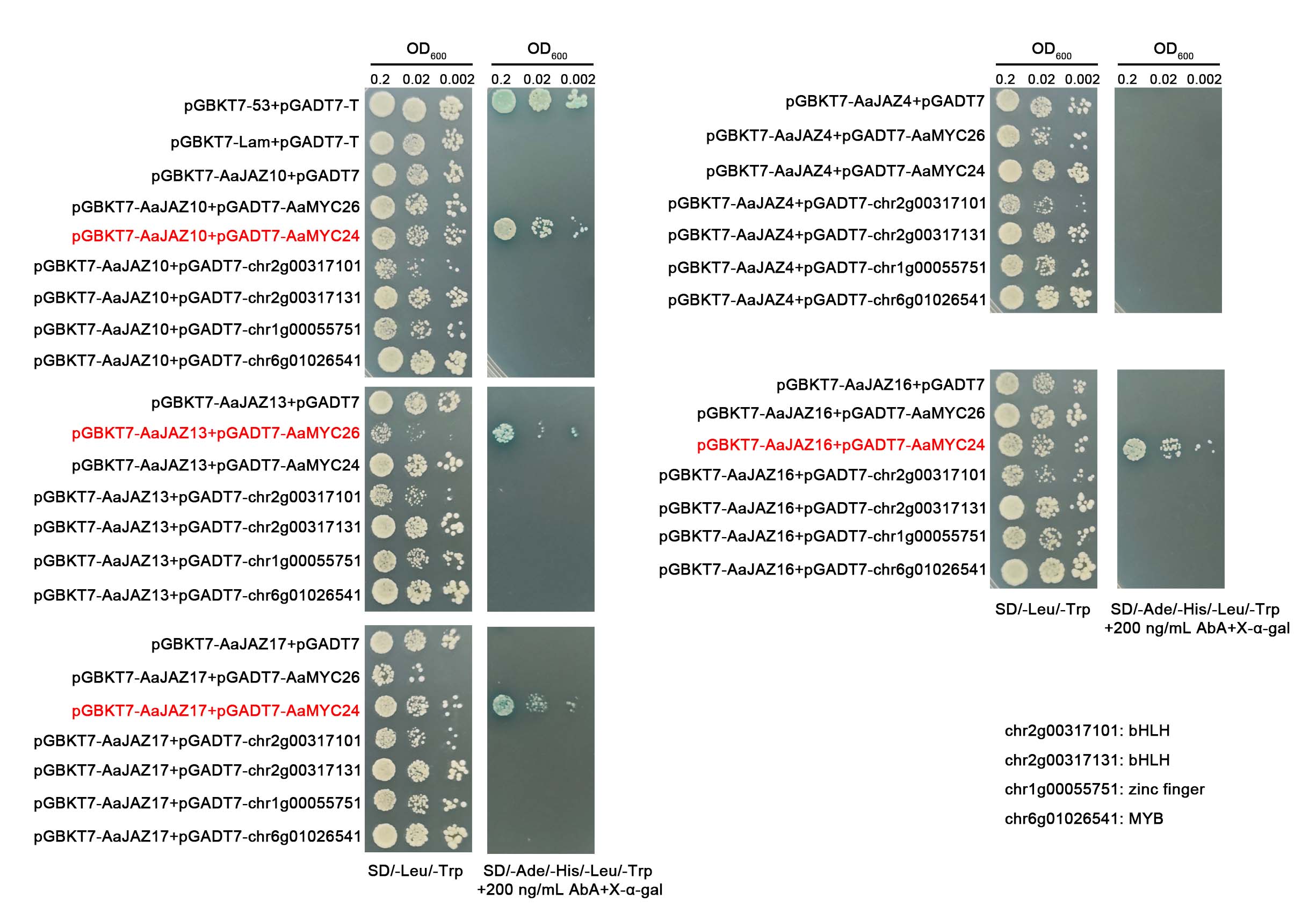


Figure S11 Protein-protein interaction by Y2H assay.
