# Supplementary material for: AaJAZ8 forms an extensive interaction network with AaJAZ proteins and two novel AaMYC transcription factors in Artemisia annua
Source: Front Plant Sci. 2026 Jun 11;17:1855917. doi: 10.3389/fpls.2026.1855917 (PMC13294869; doi:10.3389/fpls.2026.1855917)
Supplement: Supplementary file 2 [file DataSheet1.pdf]

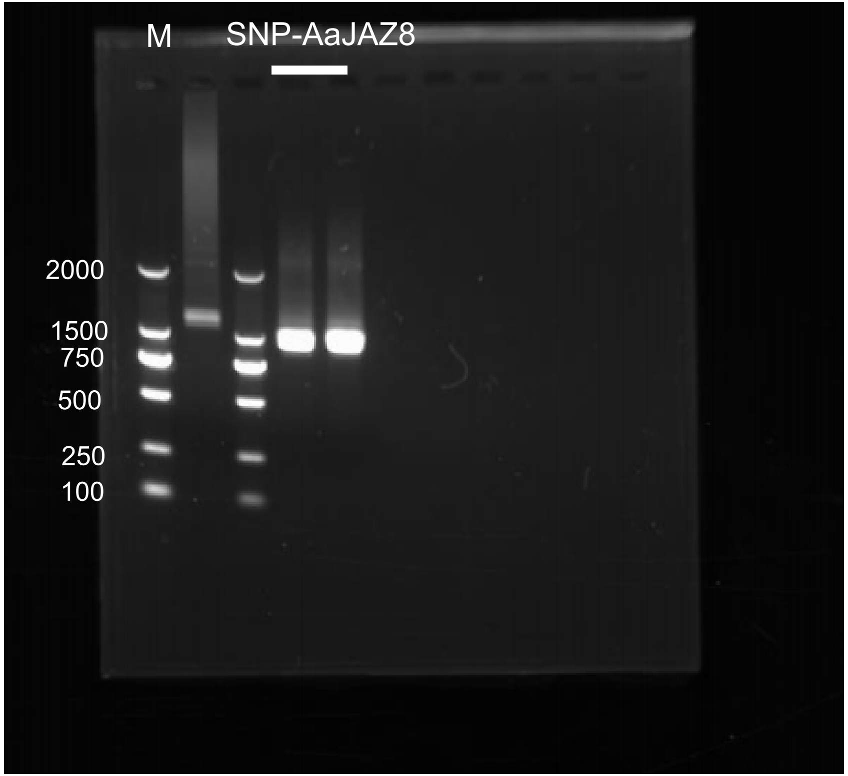
M
SNP-AaJAZ8
2000
1500
750
500
250
100

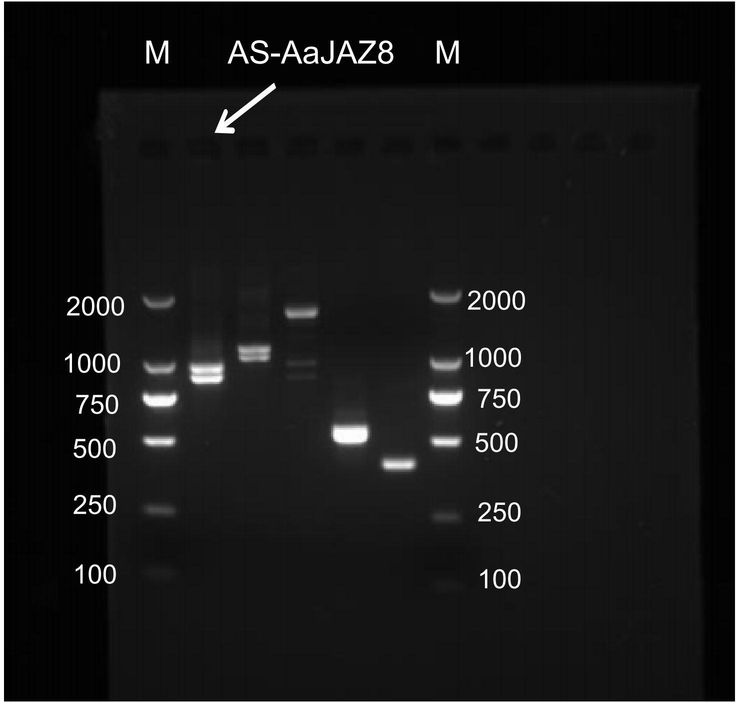

M
AS-AaJAZ8
M
2000
1000
750
500
250
100
2000
1000
750
500
250
100

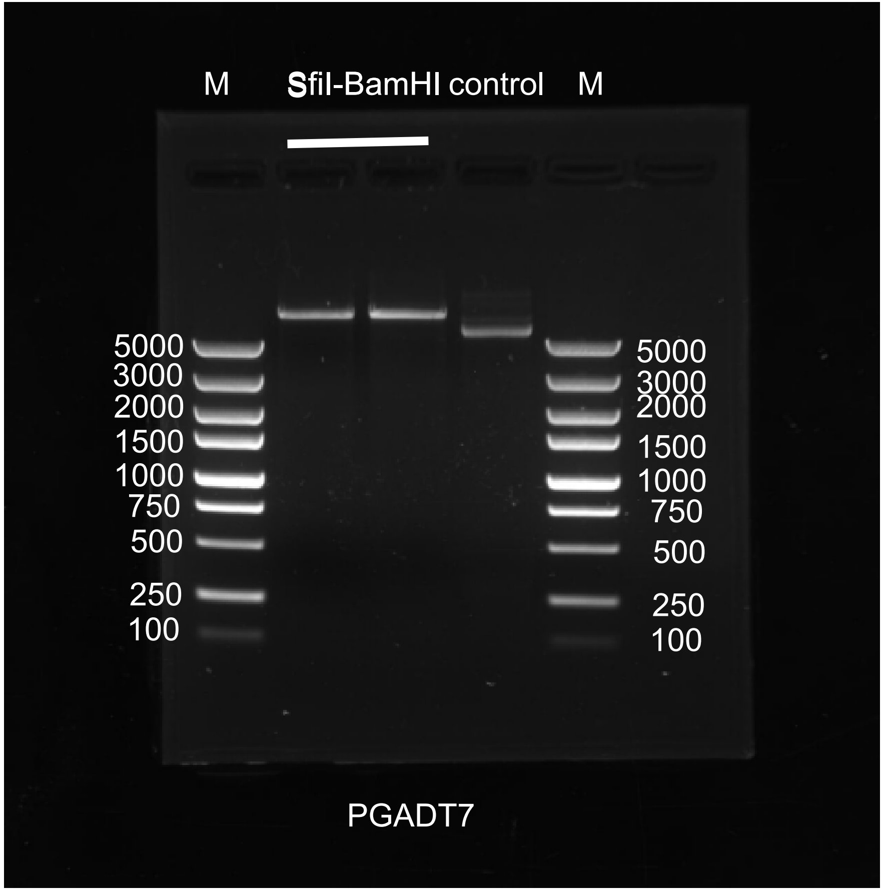
M
SfiI-BamHI control
M
5000
3000
2000
1500
1000
750
500
250
100
5000
3000
2000
1500
1000
750
500
250
100
PGADT7

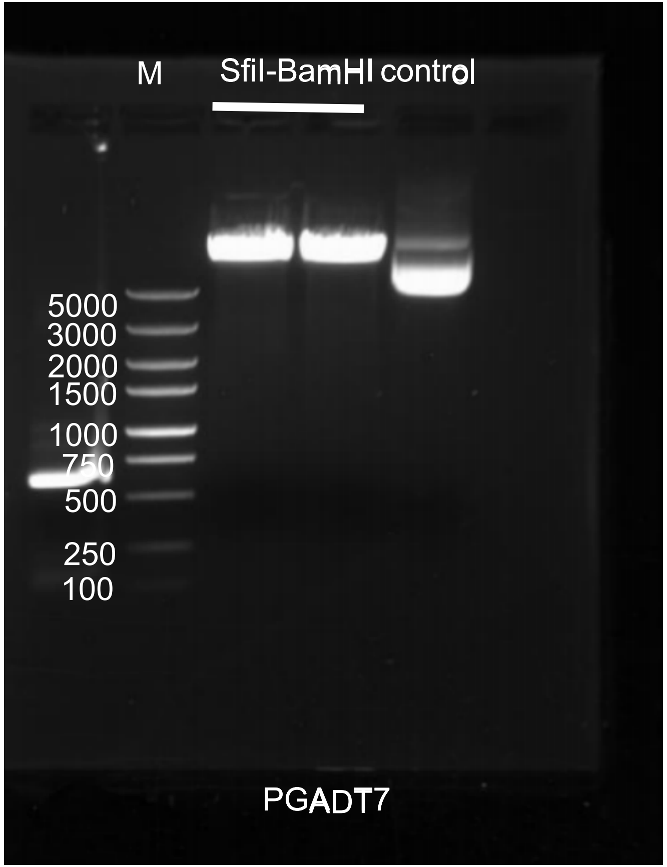
M
SfiI-BamHI control
5000
3000
2000
1500
1000
750
500
250
100
PGADT7

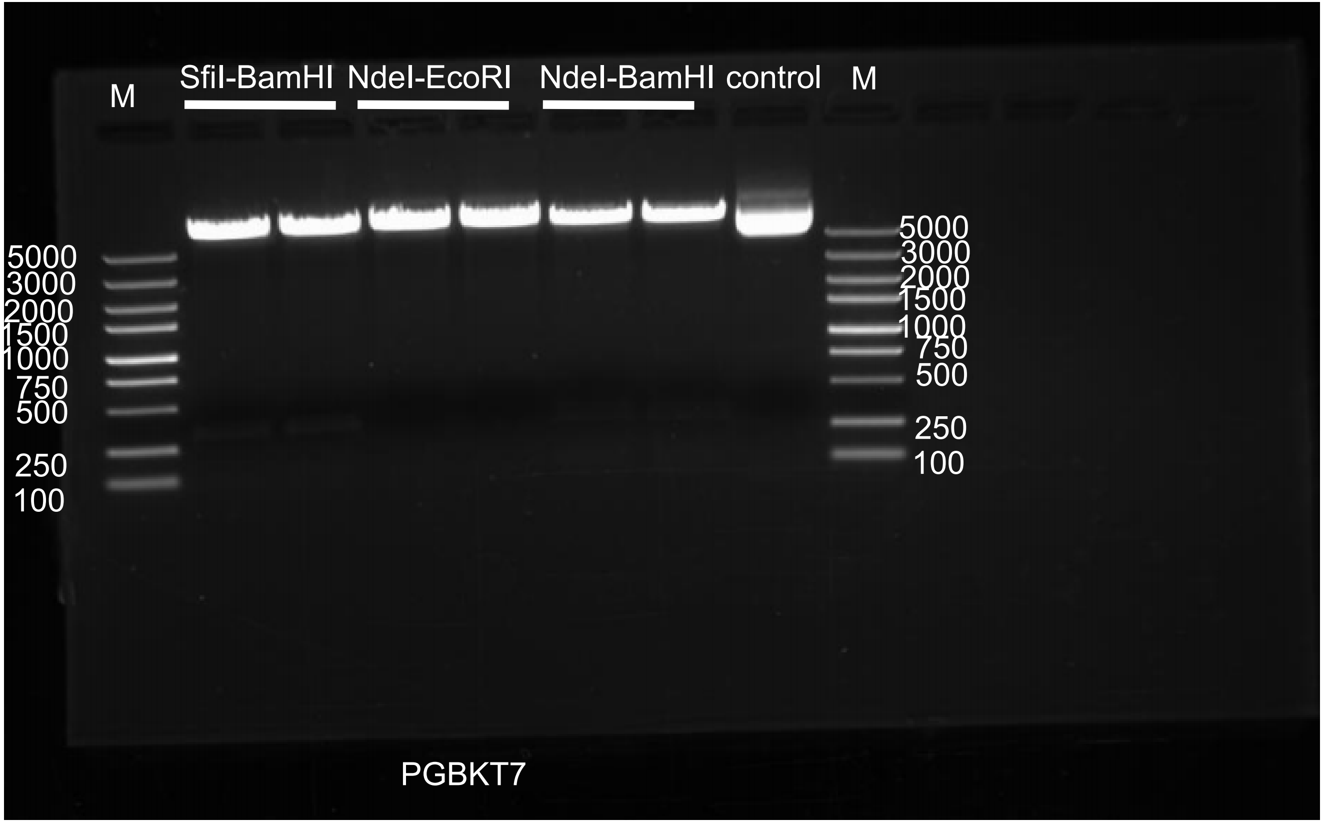
M
SfiI-BamHI
NdeI-EcoRI
NdeI-BamHI
control
M
5000
3000
2000
1500
1000
750
500
250
100
5000
3000
2000
1500
1000
750
500
250
100
PGBKT7

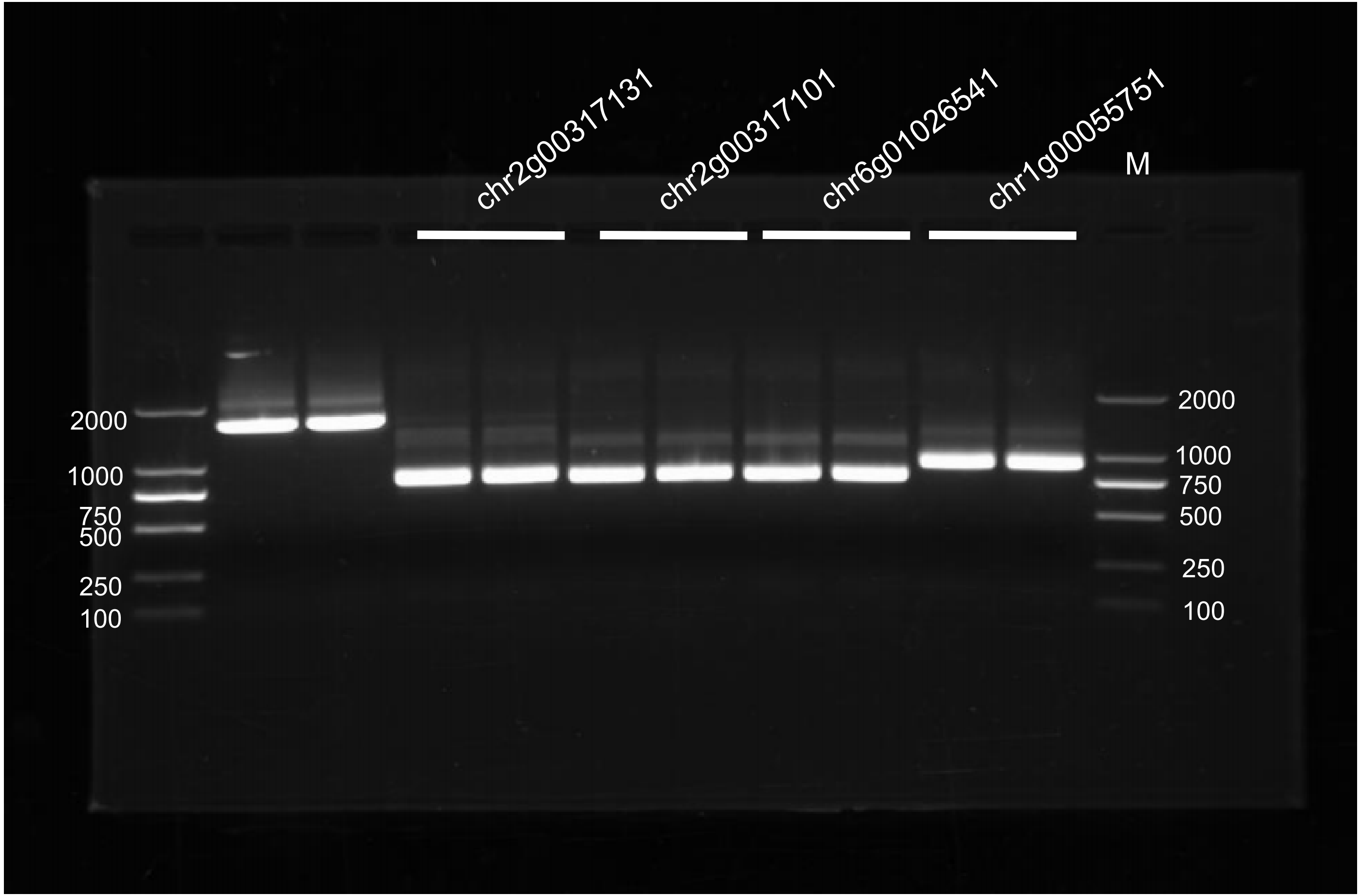

chr2g00317131
chr2g00317101
chr6g01026541
chr1g00055751
M
2000
1000
750
500
250
100
2000
1000
750
500
250
100

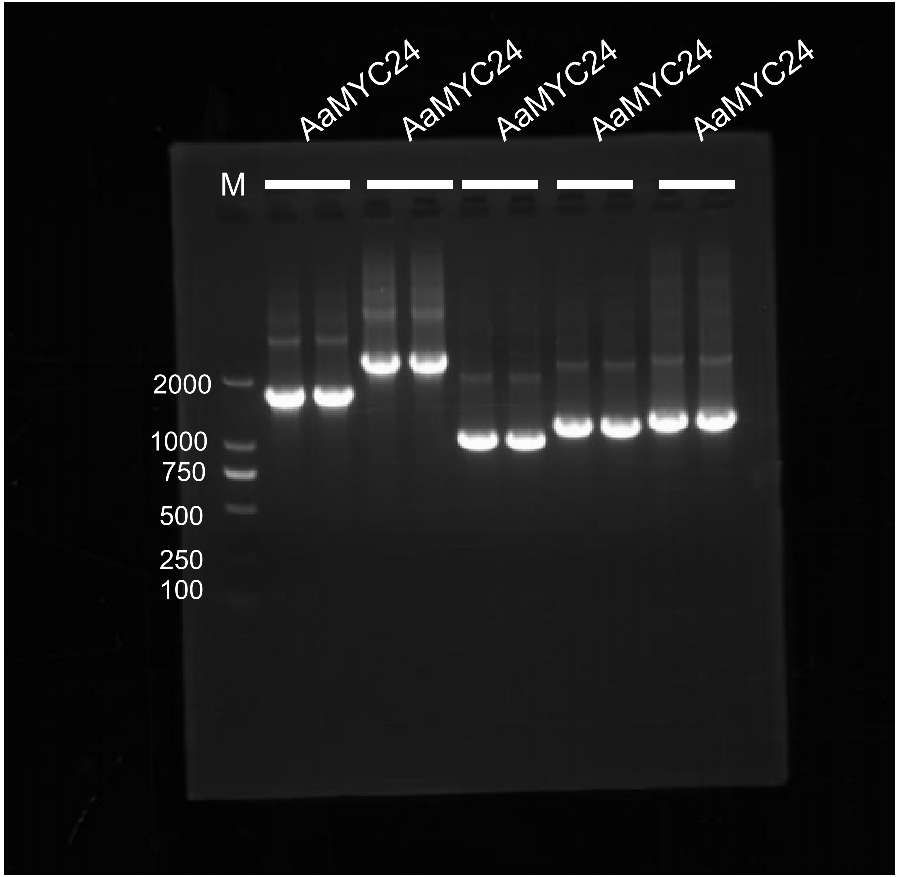
AaMYC24
AaMYC24
AaMYC24
AaMYC24
AaMYC24
M
2000
1000
750
500
250
100

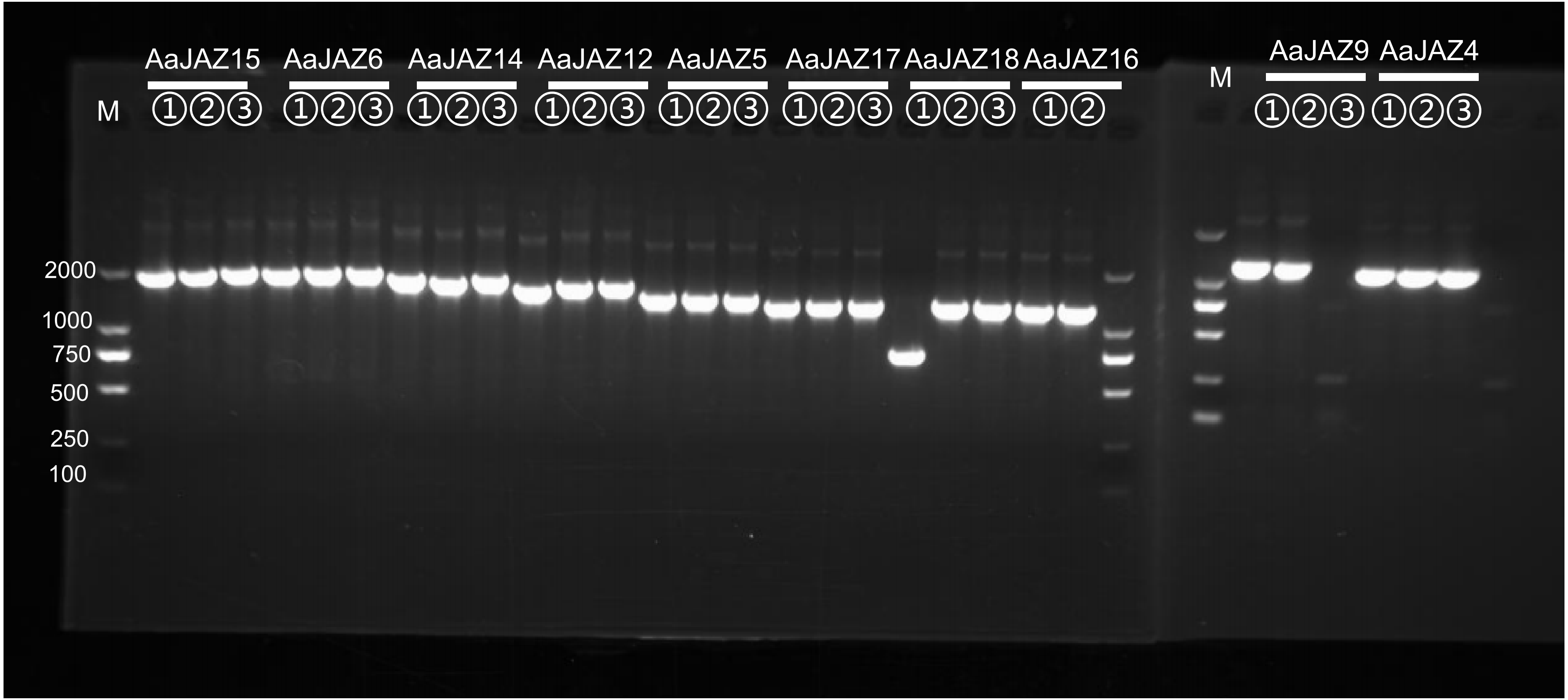

M
AaJAZ15
①②③
AaJAZ6
①②③
AaJAZ14
①②③
AaJAZ12
①②③
AaJAZ5
①②③
AaJAZ17
①②③
AaJAZ18
①②③
AaJAZ16
①②
M
AaJAZ9
①②③
AaJAZ4
①②③
2000
1000
750
500
250
100

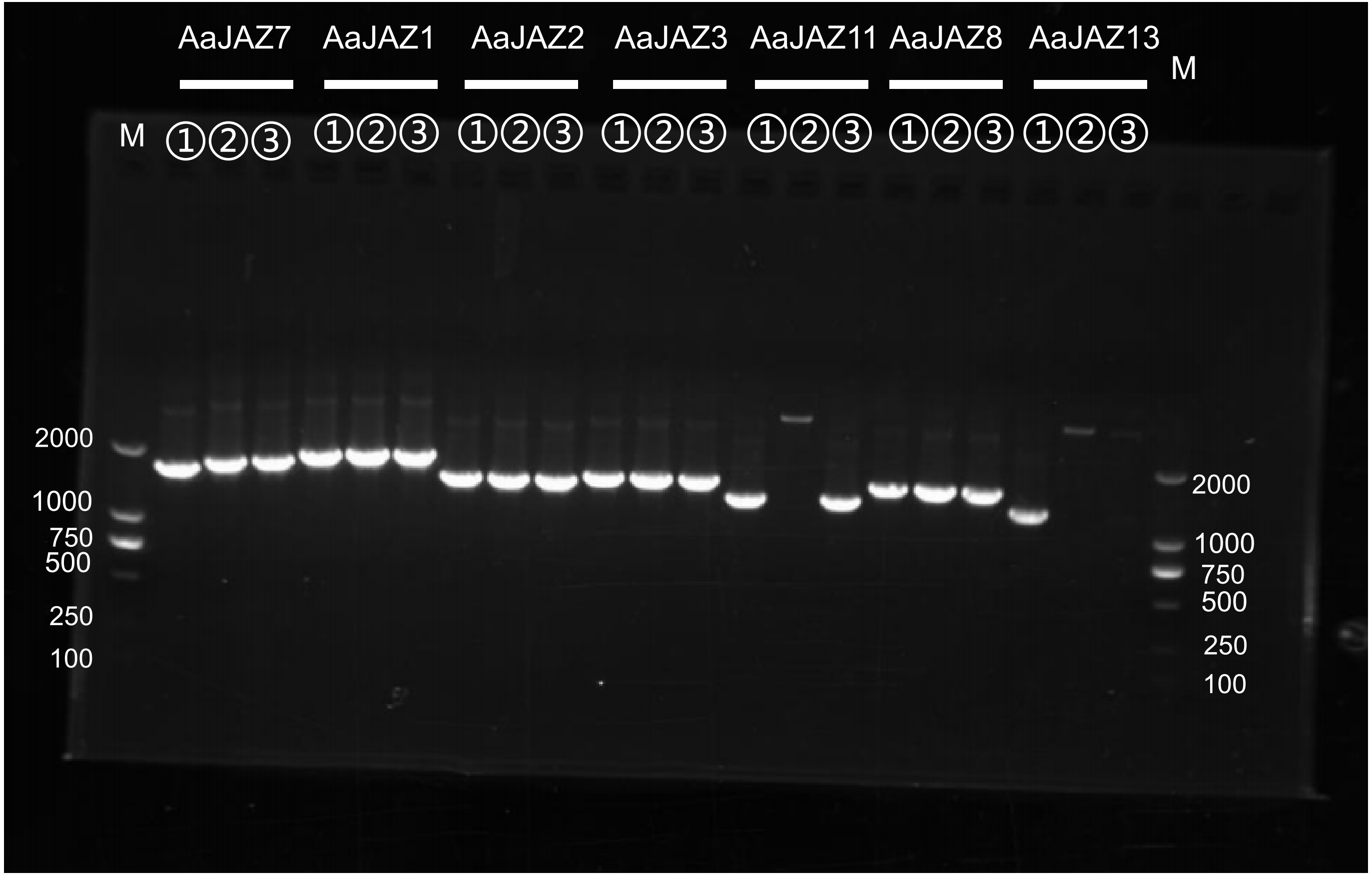

AaJAZ7
AaJAZ1
AaJAZ2
AaJAZ3
AaJAZ11
AaJAZ8
AaJAZ13
M
M
①②③
①②③
①②③
①②③
①②③
①②③
①②③
2000
1000
750
500
250
100
2000
1000
750
500
250
100

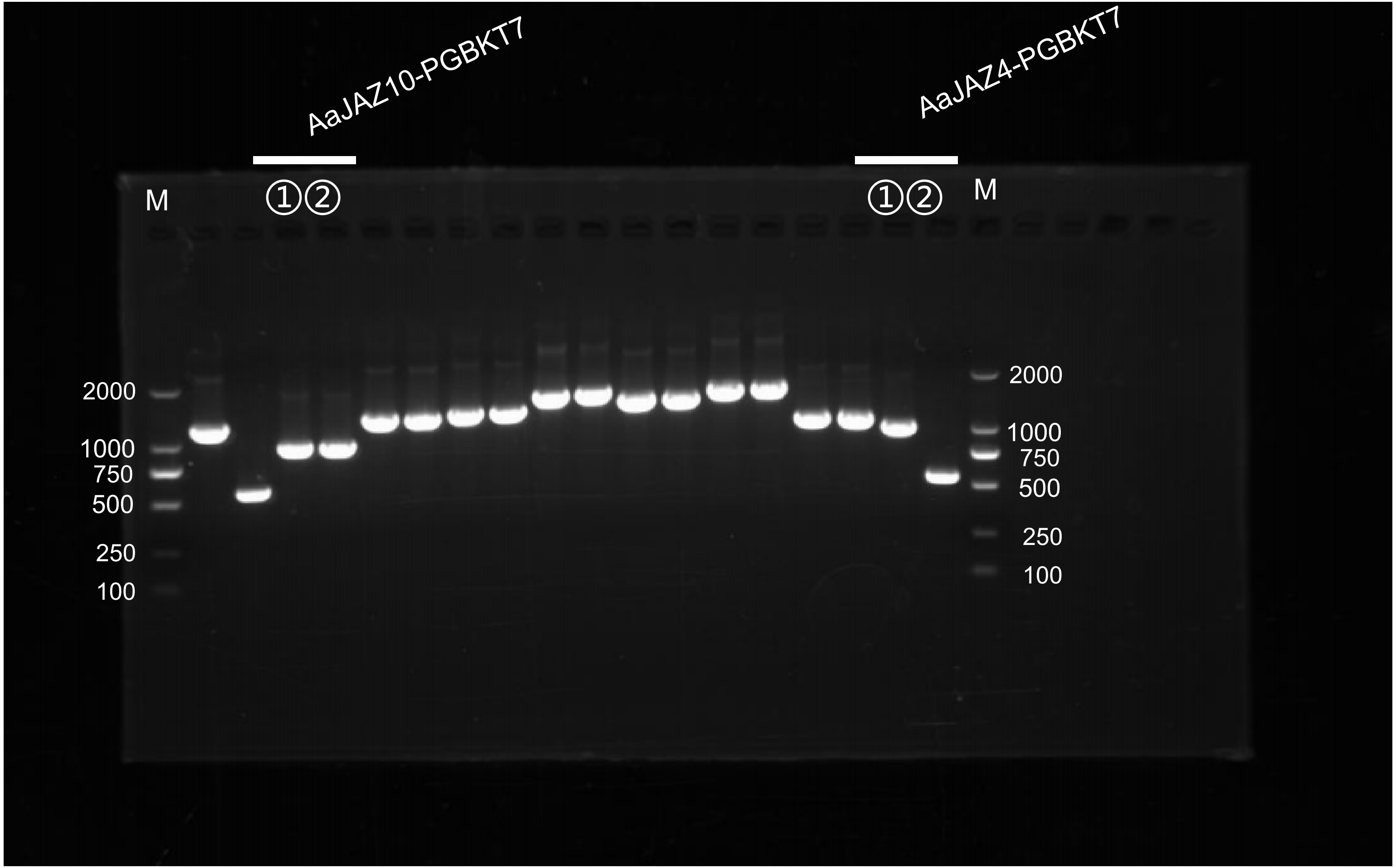

AaJAZ10-PGBKT7
AaJAZ4-PGBKT7
M
①②
①②
M
2000
1000
750
500
250
100
2000
1000
750
500
250
100

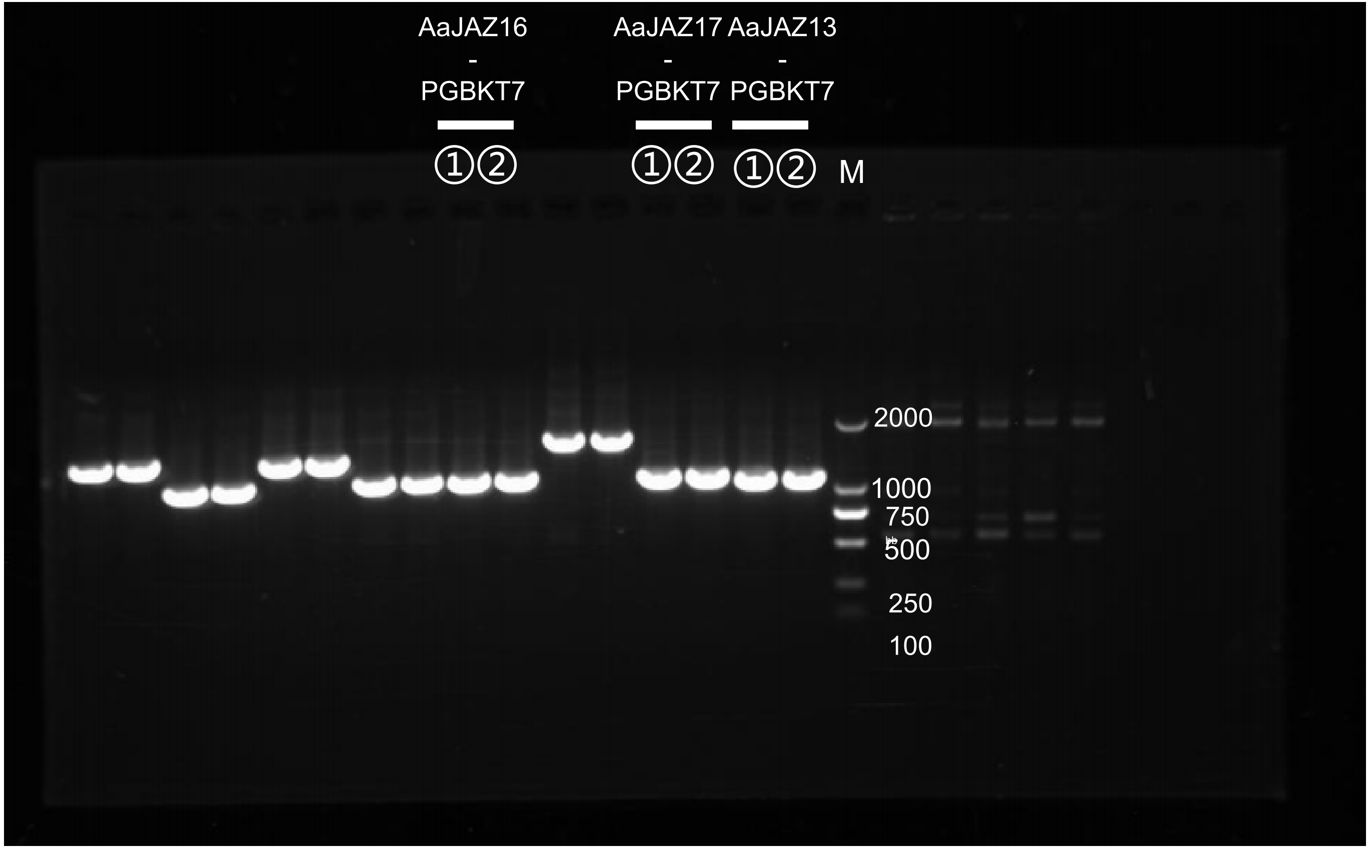
AaJAZ16 - PGBKT7
①②
AaJAZ17 - PGBKT7
①②
AaJAZ13 - PGBKT7
①②
M
2000
1000
750
500
250
100
